# Combined family-based association and linkage analyses in families affected by attention-deficit hyperactivity disorder

**DOI:** 10.1007/s00439-026-02840-7

**Published:** 2026-06-22

**Authors:** Cristina M. Justice, Kwangmi Ahn, Benjamin Jung, Luke J. Norman, Gustavo Sudre, Wendy Sharp, Marine Bouyssi-Kobar, Saadia Choudhury, Stevi Gligorovic, Paul Kundzicz, Maria T. Acosta, Philip Shaw

**Affiliations:** 1 Neurobehavioral Clinical Research Section, Social and Behavioral Research Branch, National Human Genome Research Institute, National Institutes of Health, Bethesda, MD 20892 USA; 2 Office of the Clinical Director, National Institute of Mental Health, Bethesda, MD 20892 USA; 3 King’s Maudsley Partnership, Institute of Psychiatry, Psychology and Neuroscience, King’s College London, Denmark Hill, London, SE5 8AZ UK; 4 Section on Social and Cognitive Developmental Neuroscience, National Institute of Mental Health, Bethesda, MD 20892 USA; 5 Office of the Clinical Director and Medical Genetic Branch, National Human Genome Research Institute, National Institutes of Health, Building 31, Room B1B37, 31 Center Drive, Bethesda, MD 20892 USA

## Abstract

Attention deficit hyperactivity disorder (ADHD) is one of the most prevalent and heritable of neurodevelopmental disorders. To characterize the genetic variants contributing to this heritability, we studied families with members affected by ADHD. Genome-wide array data were obtained on two cohorts: NHGRI Family Cohort (359 nuclear families,1538 individuals) and NCR Family Cohort (25 multigenerational and 132 nuclear families, 631 members). A meta-analysis of family-based association tests (FBAT) of the two cohorts identified three genome-wide significant associations, one upstream from *TFRC*, and two that were intronic to *GSDME* and to *TRIM31*, with the last gene previously implicated in ADHD. Genes associated with ADHD (MAGMA, gene-based association *P* < 0.05) overlapped with genes implicated in ADHD by prior case/control genome-wide association studies. Meta-analyses of the linkage signals identified one significant linkage region (LOD > 3) on 19p13.2-13.11 that encompassed a genome-wide significant variant in the FBAT of the NHGRI Family Cohort (rs55741253, *P* = 2.68 × 10^− 8^). Additionally, two of the five linkage regions that reached a LOD > 2 in our meta-analysis replicated significant linkages from prior studies (prior studies reporting 13 linkages with LOD > 3.0; hypergeometric test of overlapping linkage signals, *P* < 0.01). Using neuroimaging data from the NCR cohort, we found genes associated with ADHD overlapped with genes associated with the brain’s total surface area and a feature of functional connectivity, reflecting the interaction between the brain’s default mode and task positive networks. Such work begins to delineate the neural substrates of the discovered genetic associations with familial ADHD.

**Supplementary Information:**

The online version contains supplementary material available at 10.1007/s00439-026-02840-7.

## Introduction

Attention-deficit/hyperactivity disorder (ADHD) is a prevalent psychiatric disorder characterized by impairing symptoms of inattention, hyperactivity and impulsivity with heritability estimates of between 77 and 88% (Faraone and Larsson 2019). Recent landmark discoveries into common genetic variants conferring risk for ADHD have been made through genome-wide association studies (GWAS) from the Psychiatric Genomics Consortium of unrelated ADHD cases and unaffected controls (Demontis et al. 2019; 2023). Here we extend gene discovery through applying family-based association and linkage analyses to individuals from nuclear or extended multigenerational families affected by ADHD. Our family-based approach is appropriate given the high heritability of ADHD and is more resistant to issues of population stratification than those present in case-control studies of unrelated individuals (Lange et al. 2008). By identifying genomic variation associated with familial ADHD, the study can thus both complement and extend the recent GWAS findings on ADHD, in which cases were ascertained without reference to the presence or absence of family history.

Prior studies of familial ADHD have mainly used linkage analyses, mapping the segregation of ADHD with genetic loci across generations. Five studies have reported significant linkage at LOD > 3.0 for three genomic locations (5p13 (Ogdie et al. 2004; Hebebrand et al. 2006), 9q22 (Asherson et al. 2008; Romanos et al. 2008) and 17p11 (Ogdie et al. 2004; Arcos-Burgos et al. 2004) with independent replication for all signals occurring only at nominal levels of significance (LOD > 1.5). A meta-analysis data combining the results from four projects, for a total of 2,064 trios and 896 cases and 2,455 controls, did not find any genome-wide significant associations.(Neale et al. 2010).

Several ADHD studies have used a family-based association test approach. The largest involved 958 trios with probands diagnosed with ADHD and conduct disorder and reported no variants reaching genome-wide significance (Anney et al. 2008), while another study of 607 families found nominal significant to 12 SNPs (Oades et al. 2008).

Here we use both approaches- family-based association tests and linkage analyses - to examine genetic associations in two family cohorts. The first cohort, the NHGRI Family Cohort, comprises 1,538 individuals from 359 nuclear families (Acosta et al. 2008), including 423 individuals with ADHD and 1,115 unaffected individuals. The second cohort, the NCR Family Cohort, comprises 631 individuals from 25 multigenerational extended families and 132 nuclear families, including 239 individuals with ADHD and 392 unaffected individuals. Both cohorts were ascertained through families with ADHD and therefore are not representative of the general population. For context, the global prevalence of ADHD has been estimated at 7.6% in children and 5.6% in adolescents based on cross-sectional studies worldwide, which is substantially lower than the proportion observed in our cohorts due to this ascertainment strategy. Linkage and family-based association approaches can provide complementary insights: for example, while linkage analyses detect peaks that can encompass many genes, family-based associations falling within a peak can help prioritize loci or genes for further characterization. Equally, findings from linkage and family-based associations can also differ as they calculate the relationship between phenotype and genotype differently.

Once genetic associations with familial ADHD are ascertained it becomes important to consider how they might be acting on brain structure and function to give rise to symptoms. One of the cohorts in the current study had multimodal neuroimaging imaging available on many individuals, affording an opportunity to begin this process. Here, we focused on neural differences that have been robustly associated with ADHD, through mega-analytic, large scale imaging studies (Hoogman et al. 2017; 2019; Norman et al. 2022; Sudre et al. 2022). Leveraging in vivo neuroimaging, we considered both the anatomic changes most consistently tied to ADHD, and anomalies in the brain’s structural and intrinsic functional connectivity, based on a model of ADHD as being tied to disrupted interactions between key brain functional networks (Norman et al. 2022; Sudre et al. 2022). We estimated the overlap between genes associated with familial ADHD and genes associated with these key neural features. Such work is a first step towards parsing the neural substrates that may help explain associations between common genetic variation and the ADHD symptoms.

In summary, we report on family-based association and linkage analyses on two family cohorts, comprising a total of 2,169 individuals. We determine if genes emerging in our family-based study overlapped with those implicated through recent GWAS of unrelated cases and controls. Finally, we leverage neuroimaging data to begin to parse the neural substrates that might contribute to the discovered genetic associations. Although this is a small sample, compared to the number of individuals used in recent case/control studies of ADHD, it consists of linkage and association tests on a large number of extended families, which are known to find variants with larger effects than those identified through case/control associations.

## Materials and methods

### Cohorts

#### NHGRI family cohort

The main inclusion criterion for families was the presence of a child with ADHD aged between 7 and 17 years old, with at least one sibling, either affected or unaffected, ideally with both biological parents available for assessment (Acosta et al. 2008). Families with two affected parents were excluded, because such pedigrees complicate interpretation of transmission patterns and reduce clarity in distinguishing parental contribution within family-based analyses. The comorbidities listed in Table S1 were excluded since they can confound the diagnosis and introduce alternative biological causes of inattention and hyperactivity. Diagnosis of childhood ADHD was based on the clinician interview with the parent (Diagnostic Interview for Children and Adolescents-IV [DICA]) (Reich 2000). Assessment of ADHD in adults (parents and siblings) aged over 18 years was based on the Vanderbilt ADHD Diagnostic Rating Scale (VADRS) (Wolraich et al. 2003), using the cut-off of > 5 on symptom counts. For adults, a retrospective diagnosis of probable childhood ADHD was defined based on scores from the WURS (score > 35 if female and > 40 if male). Only one comorbidity, childhood oppositional defiant disorder (ODD), was sufficiently prevalent to be considered in separate FBAT analyses. Principal component analysis (PCA) performed using PLINK v1.9.0 (Purcell et al. 2007) identified 90.6% of the individuals in this cohort as white non-Hispanic (WNH). PCA analysis was performed to characterize this cohort. No individuals were excluded based on the PCA results, as the family-based tests used in this study were robust to population stratification. The study was approved by the institutional review board of the NHGRI; written informed consent was obtained from adult participants and parents.

DNA was extracted from blood and genotyped in two phases. First, 840 DNA samples consisting mainly of parent offspring trios (481 males and 359 females) were genotyped with the Global Screening Array v1.0 with Multi-Disease Component (GSA-MD v1.0) at the Broad Stanley Center for Psychiatric Disease, Broad Institute, Cambridge, MA, USA. An additional 775 samples (439 males and 336 females), consisting primarily of discordant and concordant siblings, as well as 8 replicates and 81 samples previously genotyped with the GSA v1.0, were genotyped at the Genomic Core of the NHGRI, using the GSA v3.0. The samples genotyped on GSAv1.0 and GSAv3.0 were merged, resulting in 601,123 overlapping variants available for analysis. Map position information was based on GRCh37/hg19. Any variants that were discordant among 81 replicates genotyped on both arrays were discarded. Variants were also dropped if missing rate > 5%, were unmapped, had a Mendelian inconsistency in > 1 trio, had minor allele frequency (MAF) < 5%, deviated from Hardy-Weinberg equilibrium (HWE) (*P* < 0.00001) or were discordant between duplicates. The genotypes for variants with a Mendelian inconsistency found in only one trio were zeroed out. Identity-by-descent (IBD) and Mendelian error analyses were conducted with PLINK v1.9.0 (Purcell et al. 2007). For the IBD analysis, we used a panel of variants that were not in linkage disequilibrium, and which were retained after all quality control thresholds previously mentioned were met. Four monozygotic (MZ) twins and one set of identical triplets were identified, of which one individual from each set with the highest genotyping rate was retained. MZ twins are almost identical at every variant, whether these are disease-associated or not, so keeping both MZ twins in a linkage study can lead to false positives. A total of 55 samples were removed due to IBD and/or Mendelian errors. The remaining merged set of individuals genotyped with GSAv1.0 and GSAv3.0 consisted of 1,538 individuals (877 males and 661 females) belonging to 359 informative families (Table 1). Of these 359 families, 330 met the proposal goal of enrolling a child with ADHD and at least one affected or unaffected sibling, while 29 families were composed of case-parent trios, for which the additional sibling did not pass quality control filtering.

**Table 1:** Gender, age, and comorbidity information of the NHGRI Family and NCR Family Cohorts analyzed using FBAT

	NHGRI Family	NCR
Total	1,538	631
Males (%)	877 (57.0)	363 (57.5)
Females (%)	661 (43.0)	268 (42.5)
Average age (standard deviation)	28 (17)	25 (18)
Number of families	359	157
Median family size (range)	4.28 (3–18)	4.02 (2–25)
ADHD (%)	423 (27.5)	239 (37.9)
Race (%)		
White	1442 (93.8)	513 (81.3)
African American	23 (1.5)	49 (7.8)
Asian	26 (1.7)	21(3.3)
Native American	10 (0.7)	
> 1 Race	37 (2.4)	38 (6.0)
Other (%)		3 (0.5)
Not specified (%)		7 (1.1)
Ethnicity (%)		
Hispanic	58 (3.8)	49 (7.8)
Non-Hispanic	1480 (96.2)	579 (91.8)
Not specified		3 (0.5)
Comorbidities (%) for individuals < 19 years of age		
Oppositional Defiant Disorder (ODD)	205 (28.7)	25 (7.0)
Conduct Disorder	16 (2.2)	1 (0.3)
Major Depressive Disorder	4 (0.6)	16 (4.3)
Mania	2 (0.3)	
Dysthymic Disorder	8 (1.1)	
Separation Anxiety	5 (0.7)	1 (0.3)
Generalized Anxiety	25 (3.5)	12 (3.3)
Obsessive Compulsive Disorder	11 (1.5)	1 (0.3)
Post-traumatic stress disorder	2 (0.3)	
ADHD + ODD diagnosis	184 (42.8)	31 (8.5)
Comorbidities (%) for individuals ≥ 19 years of age		
Screened Positive for Anxiety or Depression	116 (14.5)	53 (19.0)
Dysthymic Disorder		1 (0.5)

#### NCR family cohort

The Neurobehavioral Clinical Research (NCR) Family Cohort comprised both extended and nuclear families- demographic details in Table 1. The inclusion criteria for the extended families were (1) the presence of second-, third-, or higher-degree relatives and (2) a diagnosis of ADHD in at least 25% of family members. For nuclear families, the main inclusion criterion was at least two siblings, one with ADHD. The study was approved by the institutional review board of the NHGRI; written informed consent was obtained from adult participants and parents; children gave written assent. For children, diagnosis of ADHD was through the parental Diagnostic Interview for Children and Adolescents-IV (DICA) (Reich 2000). For adults, the Conners’ Adult ADHD Diagnostic Interview for DSM-IV(CAADID) (Epstein, Johnson, and Conners 2001; Conners and Staff 2004) was used to ascertain both adult and childhood history of ADHD symptoms. Presence of other psychiatric diagnoses was established through the Structured Clinical Interview for DSM-IV-TR Axis I Disorders, Research Version, Patient Edition (First et al. 2002). All interviews were conducted by 2 experienced clinicians (W.S. and P.S.) with interrater reliabilities of κ > 0.9. The main exclusion criteria were an IQ less than 70 (Wechsler 2011), neurologic disorders affecting brain structure, or psychotic disorders. Further details on data collection, genotyping process and phenotype information collected are detailed elsewhere (Sudre et al. 2017, 2020, 2021).

The samples were genotyped on the Infinium OmniExpressExome-8v1. Quality control steps included dropping variants if genotyping call rate < 95%, were not mapped, had MAF < 5%, were discordant in duplicates (21 duplicates) or HWE *P* < 0.00001. Mendelian inconsistencies were detected using PLINK v1.9.0 (Purcell et al. 2007) and PEDSTATS (Wigginton and Abecasis 2005). Variants with > 1 Mendelian inconsistency were dropped; if inconsistency found in only one trio, the genotypes for corresponding variant and trio were zeroed out. IBD analyses were conducted with PLINK v1.9.0 (Purcell et al. 2007). Eighteen monozygotic twins were identified, of which the twin from each pair with the higher missing genotyping rate was dropped. One family and one sib-pair were dropped due to IBD inconsistencies. For the duplicated samples, we dropped one with higher missing genotyping rate. The final sample consisted of 631 individuals (268 females and 363 males) from 157 families (Table 1), of which 73.2% were identified as WNH though PCA using PLINK v1.9.0 (Purcell et al. 2007). No samples were excluded based on PCA results, since the family-based tests used are immune to population stratification bias.

For the NCR Family Cohort only, neuroanatomic data (acquired using T1-weighted images on a 3 T Siemens scanner) was available on 370 subjects that passed quality control. We extracted neuroanatomic features that have been most consistently associated with ADHD through multi-site mega-analytic, cross-sectional studies: total brain volume, striatal and amygdala volume reduction (Hoogman et al. 2017), right lateral orbitofrontal and superior frontal cortical surface area reductions, and a thinner right fusiform and precentral cortex (Hoogman et al. 2019). In addition, diffusion tensor imaging that passed quality control was available on 345 subjects from which we extracted measures of the microstructure (fractional anisotropy) of the two tracts (inferior longitudinal fasciculi and left uncinate fasciculus) that were robustly associated with ADHD in a recent mega-analysis (Sudre et al. 2022). Finally, 359 subjects had functional connectivity metrics from resting state fMRI- from which we extracted a measure of the atypical connectivity between the default mode network (that characterizes interoceptive processing) and networks supporting attention, cognitive control and motor planning and execution, as we found these interactions to be most robustly associated with ADHD (Norman et al. 2022). Details of acquisition and quality control in Supplemental Methods. There was thus a total of 20 neural measures.

### Imputation analysis

The two cohorts were genotyped on different arrays with less than 100,000 shared variants. To increase overlap the cleaned genotypes were fed to the Mimimac4 software (Das et al. 2016) on the University of Michigan Imputation server v.1.2.4 (https://imputationserver.sph.umich.edu), using the 1000G Phase 3 v5 GRCh37/hg19 reference panel and the European (EUR) population. Genotypes were phased prior to imputation using Eagle v2.4 (Das et al. 2016) and filtered by imputation quality score Rsq > 0.3. Imputed variants were dropped prior to analyses if they had an HWE *P* < 0.00001, MAF < 5%, or any Mendelian inconsistency detected by using either PLINK v1.9.0 (Purcell et al. 2007) or PEDSTATS (Wigginton and Abecasis 2005)). For association and linkage analyses, we selected for analysis the 244,195 variants initially genotyped in NHGRI Family Cohort that had either genotyped or imputed corresponding variants in the NCR Family Cohort that passed quality control.

### Family-based association test (FBAT)

The GWAS was carried out using the Family-Based Association Test (Rabinowitz and Laird 2000; Laird et al. Laird 2000). The FBAT is a generalization of the TDT (Spielman, McGinnis, and Ewens 1993), which measures the rate of transmission of an allele from the heterozygous parents to an affected offspring. FBAT (v2.0.2) breaks the extended pedigrees into nuclear families and can deal with missing parental genotypes and phenotypes, while testing for associations in the presence of linkage. Association results are the sum of the statistics across the independent families, assuming an additive model with a null hypothesis of no linkage or association. The minimum number of informative families used for calculating the test statistic was set at 10. FBAT was run independently on the 359 families in the NHGRI Family Cohort and the 157 families from the NCR Family Cohort. We performed a meta-analysis of summary statistics from FBAT of the two cohorts with METAL (http://www.sph.umich.edu/csg/abecasis/metal/index.html) using a fixed-effects meta-analysis weighted by sample number for each study and testing for between-study heterogeneity. A heterogeneity p-value (*P*_het_) > 0.1 indicated homogeneity of the effect size across both cohorts.

### Linkage analysis

Multipoint non-parametric linkage analysis was performed using MERLIN v1.1.2 (Abecasis et al. 2002) to test for linkage between the diagnosis of ADHD and the array variants. The genetic distances (in cM) were assumed to be a linear function of the map distance (in Mb), and the physical distances were converted to map units (1 cM = 1 Mb). Prior to linkage analysis, variants were pruned for linkage disequilibrium (LD) with PLINK v1.9.0 (Purcell et al. 2007) using a pairwise analysis of 5 variants at a time in a 50 kb window and a pairwise r^2^ threshold < 0.2. This resulted in 62,239 autosomal variants with an MAF > 5% available for analysis. Only families with two parents and at least two offspring (380 families, 1831 individuals) were analyzed for linkage, of which 330 families (1451 individuals; family size range: 4–18) were from the NHGRI Family Cohort and 50 families (380 individuals; family size range: 4–18) were from the NCR Family Cohort. Allele frequencies were estimated from the founders.

The non-parametric linkage statistic p-values for Whittemore and Halpern (Whittemore and Halpern 1994), and the Kong and Cox logarithm of odds (LOD) scores (Kong and Cox 1997) were calculated for each variant, as implemented in MERLIN (Abecasis et al. 2002). The NHGRI Families and NCR cohorts were analyzed separately. A meta-analysis of the cohorts, using the Whittemore and Halpern p-values, was performed using METAL(http://www.sph.umich.edu/csg/abecasis/metal/index.html).

A LOD score > 3 was used as the threshold for significant evidence of linkage, while a LOD > 2 was selected as suggestive evidence of linkage, with the candidate region set at ± 1 mega base (Mb) from the linkage peaks. Although more stringent thresholds have been proposed for genome-wide linkage analyses (e.g., LOD ≥ 3.3; Lander and Kruglyak 1995), these thresholds may be too conservative for complex traits and studies with smaller sample sizes. Sawcer et al. (1997), using simulations based on their specific genome-wide linkage scan, estimated that a LOD score of 3.2 corresponded to a genome-wide significance level of 5%, demonstrating that significance thresholds depend on study design. Here we applied a LOD threshold of > 3 to balance statistical power with control of false positives.

There was a total of 13 candidate linkage regions with LOD > 3.0 reported in the literature, see Table S3. The significance of the overlap of linkage signals from our meta-analysis with the previously reported linkage regions was determined by calculating the hypergeometric probability (https://molbiotools.com/math_calculators/hypergeometric.html), setting the total population size to 400, equal to the number of chromosomal bands in the genome (Rosenberg and Rosenberg 2012).

### SNPs annotation

We performed ANNOVAR (http://annovar.openbioinformatics.org/) (Wang et al. 2010) gene-based annotation using Ensembl genes to annotate SNPs within FUMA (Watanabe et al. 2017).

### Gene-based association and gene set enrichment analysis

We conducted gene-based association analyses using the summary statistics from the FBAT of the larger NHGRI Family Cohort and from the meta-analysis of both cohorts. MAGMA v.1.08 (de Leeuw et al. 2015) (http://ctg.cncr.nl/software/magma) was used to assess the aggregated genetics effects within a locus across all protein-coding genes in the genome, declaring exome-wide significant genes at *P* < 3.08 × 10^− 6^ (0.05/16192 genes). Gene length boundaries were defined as 10 kilobases (kb) upstream and downstream from start and stop site, respectively, to include regulatory elements. The NCBI 37.3 build was used to assign the genetic variants to each gene.

Genes associated with ADHD, either through FBAT or by MAGMA gene-based association to conduct gene-set enrichment analyses, were fed into MAGMA’s competitive gene-set analysis facility using the GENE2FUNC module integrated in FUMA. Gene sets in MAGMA were obtained from the MSigDB v.7.0 for “Curated gene sets” and “GO terms”, and from reported genes from the GWAS-catalog.

Gene set enrichment analysis were also conducted using the R software package WebGestaltR (0.4.3) for Biological Process, Molecular Function, and Cellular Components. In addition, we created curated gene-sets from GWAS and transcriptome-wide association study (TWAS) for ADHD, autistic spectrum disorder, schizophrenia, and bipolar affective disorder (curated gene sets listed in Table S2). The enrichment analysis was performed using the hypergeometric test. Input was the summary statistics from MAGMA gene-based associations. For both sets of gene-set enrichment analyses, false discovery rate (FDR)-adjusted *q* values were generated using Benjamini and Hochberg’s method to account for multiple testing.

Using the GeneOverlap R package (v3.12), Fisher’s exact test was tested for overlap between genes associated with the clinical and with the brain phenotypes in the NCR Family Cohort.

### Polygenic risk score (PRS) analyses

We assessed if polygenic risk scores (PRS) for ADHD, derived from GWAS of unrelated cases and controls (the summary data from the Psychiatric Genetics Consortium (PGC) ADHD GWAS (Demontis et al. 2019)) was also associated with ADHD within our family cohorts. For this analysis, we focused on only samples of European ancestry based on genetic principal components (PC-Air (Conomos, Miller, and Thornton 2015)). We used SNP effect sizes estimates (20,183 ADHD cases and 35,191 control) from PGC summary data (https://pgc.unc.edu). Given the genetic overlap between ADHD and other disorders, we also considered polygenic risk for autistic spectrum disorders, obsessive compulsive disorder, Tourette’s syndrome, bipolar affective disorder and schizophrenia, derived from summary GWAS data from the PGC (Grove et al. 2019; International Obsessive Compulsive Disorder Foundation Genetics and Studies 2018; Yu et al. 2019; Mullins et al. 2021; Pardinas et al. 2018). After clumping, PRS were generated using PRSice-2 v2.3.5 (Choi and O’Reilly Choi 2019) for each sample at *P* value thresholds of: 1, 0.5, 0.05, 0.04, 0.03, 0.02. 0.01, 0.005, 0.001, 1 × 10^− 4^, 1 × 10^− 5^, 5 × 10^− 8^. All PRS values were normalized before analyzing. The association between PRS and ADHD status was then tested using a generalized linear mixed model implemented in the GMMAT package (Chen et al. 2016). In this model, PRS was included as a fixed-effect predictor along with age, sex, and ancestry principal components, while family structure was modeled as a random effect using a genetic relationship matrix (GRM) calculated from pruned genotype data using PLINK v1.9.0 (Purcell et al. 2007). The *glmm.wald* function was used to perform association testing.

The number of principal components retained for each cohort was determined based on the proportion of genetic variance explained, with the minimum number of PCs selected to capture at least 85% of the variance (resulting in five PCs for the NHGRI Family Cohort and three for the NCR Family Cohort). To account for multiple testing across the p-value thresholds evaluated within each PRS analysis, false discovery rate (FDR)-adjusted p-values were calculated using the Benjamini–Hochberg procedure.

## Results

### Association results

We considered 1,538 individuals [877 males; 661 females; mean age 28 (17 SD); age range 4–78] from 359 families [median size 9.5, range 3–18] in the NHGRI Family Cohort (Table 1). Nineteen SNPs were associated with ADHD at a genome-wide level of significance *P* < 5 × 10^− 8^ (Figure S1a, Table 2).

**Table 2:** Genome-wide significant (*P* < 5 × 10^−8^) results for association analysis of the NHGRI family cohort

CHR	BP	SNP	A1	A2	MAF	*P*-values	Location	Gene
3	195,838,160	rs77176301	C	T	0.059	2.95E-11	upstream	*LINC00885*,* TFRC*
4	111,707,130	rs62337211	A	C	0.15	6.17E-10	intergenic	*PITX2*,* MIR297*
4	154,603,296	rs2405433	T	C	0.19	1.87E-13	upstream; downstream	*TLR2*,* LOC100419170*,* RNF175*,* TMEM131L*
6	30,036,333	rs17187693	C	G	0.29	4.81E-08	intronic	*PPP1R11*
6	30,074,086	rs115147572	A	C	0.11	1.98E-10	intronic	*TRIM31*
6	31,045,554	rs9378152	G	C	0.13	4.43E-08	upstream; downstream	*PSORS1C1*,* C6orf15*,* CDSN*,* HCG22*,* MUC22*
7	7,223,618	rs60600046	G	A	0.06	1.52E-10	Intronic	*C1GALT1*
7	24,783,987	rs61241329	A	C	0.07	5.95E-10	intronic	*GSDME*
8	77,927,157	rs536249779	T	G	0.12	7.81E-10	upstream	*MIR3149*,* PEX2*
9	14,246,998	rs10961448	C	A	0.08	4.25E-08	intronic	*NFIB*
10	2,742,533	rs7911061	A	G	0.16	2.22E-08	intergenic	*LINC02645*,* LOC101927824*
14	52,778,656	rs5003100	A	G	0.08	1.92E-13	upstream; downstream	*PTGER2*,* PTGDR*
14	93,263,982	rs17128572	C	G	0.16	4.58E-10	exonic	*GOLGA5*
18	59,994,265	rs8087597	G	A	0.15	3.82E-10	intronic	*TNFRSF11A*
18	60,024,128	rs58112300	C	T	0.06	1.79E-09	intronic	*TNFRSF11A*
19	39,993,470	rs55741253	T	A	0.24	2.68E-08	exonic	*DLL3*
20	6,697,162	rs34169461	T	A	0.11	4.80E-13	downstream	*LINC01713*
20	8,817,556	rs58568270	G	A	0.1	5.61E-15	intronic	*PLCB1*
21	35,715,023	rs73902815	C	T	0.06	1.36E-11	upstream	*KCNE2*,* SMIM11A*,* SMIM11B*

*CHR* chromosome, *BP* base pair position (GRCh37/hg19), *A1* minor allele in NHGRI Family Cohort, *A2* major allele in NHGRI Family Cohort, *MAF*^*1*^ minor allele frequency estimated from 1106 NHGRI Family Cohort founders

**Fig. 1:**
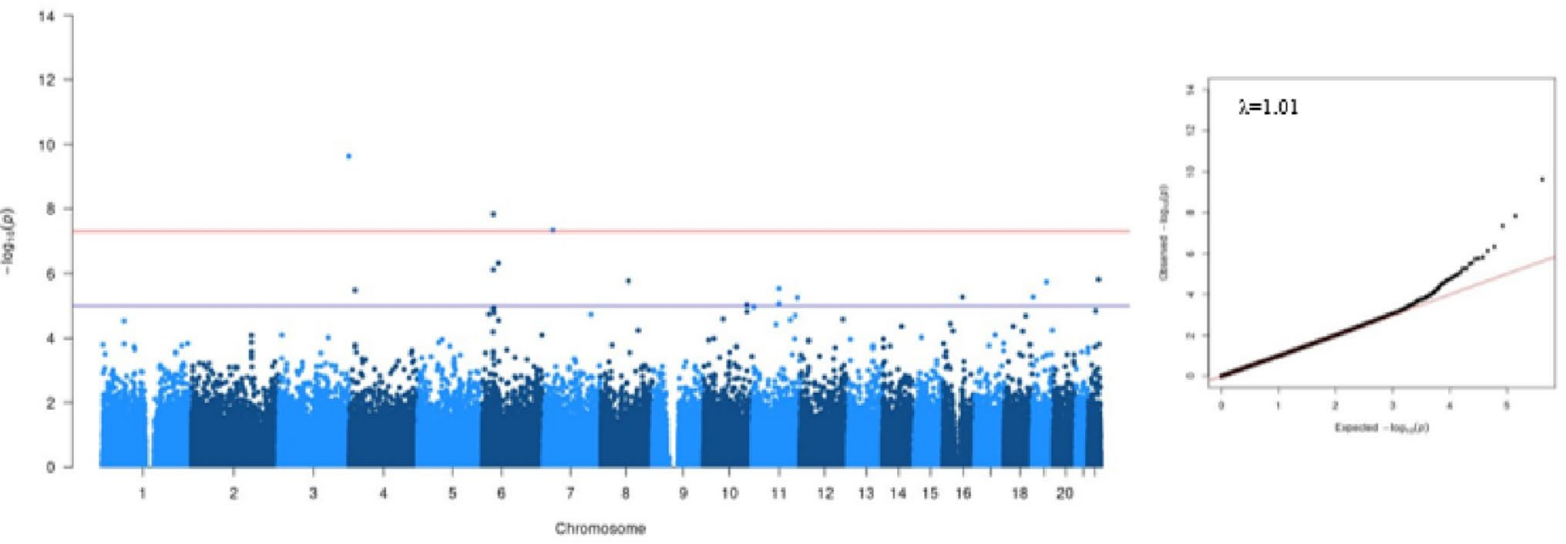
Manhattan and quantile-quantile (QQ) plots meta-analysis of FBAT results of NGRHI Family and NCR Family Cohorts. The horizontal blue line corresponds to *P* < 1 × 10^− 5^, while the horizontal red line corresponds the genome-wide significant threshold of *P* < 5 × 10^− 8^

The NCR Family Cohort consisted of 631 individuals [363 males; 268 females; mean age 25 (18 SD); age range 5–88] in 157 families [25 multigenerational, extended and 132 nuclear families, median family size 4, range 2–25 members]. No genome-wide significant associations with ADHD emerged in the NCR Family Cohort and no genome-wide significant variant in the NHGRI Family Cohort was significantly associated with ADHD in the NCR Family Cohort (Figure S1b). However, given the similarity in study design, we combined the cohorts meta-analytically, retaining the 258,399 overlapping variants that survived quality control procedures. Three of the 19 SNPs that had genome-wide significant associations with ADHD in the NHGRI Family Cohort also attained genome-wide significance (*P* < 5 × 10^− 8^) in the meta-analysis (Fig. 1): rs77176301, upstream from *TFRC*; rs115157572, intronic to *TRIM31*; and rs61241329, intronic to *GSDME*. In addition, twelve of the significant SNPs in the NHGRI Family Cohort were borderline significant in the meta-analysis (*P* < 1 × 10^− 5^) (Table 3, Figure S2), of which eight had homogeneous effects in both cohorts (*P*_het_ > 0.1). Several of the variants in Table 3 show evidence of heterogeneity (Pₕₑₜ < 0.05), indicating differences in effect sizes between cohorts.

**Table 3:** Meta-analysis results (*P* < 1 × 10^−5^) of the NHGRI family and NCR family cohorts

CHR	BP	SNP	A1	A2	MAF^1^	MAF^2^	*P*	*P*_het_	Function	Gene
3	195,838,160	rs77176301	C	T	0.06	0.05	2.32E-10	0.021	intergenic	*TFRC*,* LINC00885*
4	15,934,723	rs2532098	T	G	0.35	0.38	3.32E-06	0.866	intergenic	*CD38*,* FGFBP1*
6	30,036,333	rs17187693	C	G	0.3	0.06	7.66E-07	0.0194	intronic	*PPP1R11*
6	30,074,086	rs115147572	A	C	0.11	0.07	1.47E-08	0.0037	intronic	*TRIM31*
6	44,160,786	rs9462975	C	A	0.08	0.06	4.83E-07	0.4503	intergenic	*CAPN11*,* MYMX*
7	24,783,987	rs61241329	A	C	0.07	0.05	4.59E-08	0.0034	intronic	*GSDME*
8	77,478,539	rs28727938	C	G	0.17	0.06	1.72E-06	0.0593	intergenic	*LINC01111*,* ZFHX4-AS1*
10	121,314,587	rs116618532	G	A	0.07	0.05	9.41E-06	0.3783	intergenic	*RGS10*,* TIAL1*
11	74,137,551	rs612040	A	C	0.45	0.46	2.94E-06	0.1356	intergenic	*MIR548AL*,* KCNE3*
11	74,153,008	rs10793085	T	C	0.46	0.47	8.59E-06	0.2894	intergenic	*MIR548AL*,* KCNE3*
11	126,421,415	rs59826922	A	G	0.06	0.08	5.65E-06	0.7473	intronic	*KIRREL3*
16	54,032,493	rs11646260	G	A	0.06	0.05	5.41E-06	0.0353	intronic	*FTO*
19	2,558,131	rs78619038	G	A	0.18	0.21	5.38E-06	0.1617	intronic	*GNG7*
19	39,993,470	rs55741253	T	A	0.24	0.09	1.84E-06	0.0031	exonic	*DLL3*
22	46,614,274	rs1800206	C	G	0.16	0.06	1.53E-06	0.4838	exonic	*PPARA*

*CHR* chromosome, *BP* base pair position (GRCh37/hg19), *A1* minor allele in NHGRI Family Cohort, *A2* major allele in NHGRI Family Cohort, *MAF*^*1*^ minor allele frequency estimated from 1106 NHGRI Family Cohort founders, *MAF*^*2*^ minor allele frequency estimated from 208 NCR Cohort founders

### Linkage results

**Fig. 2:**
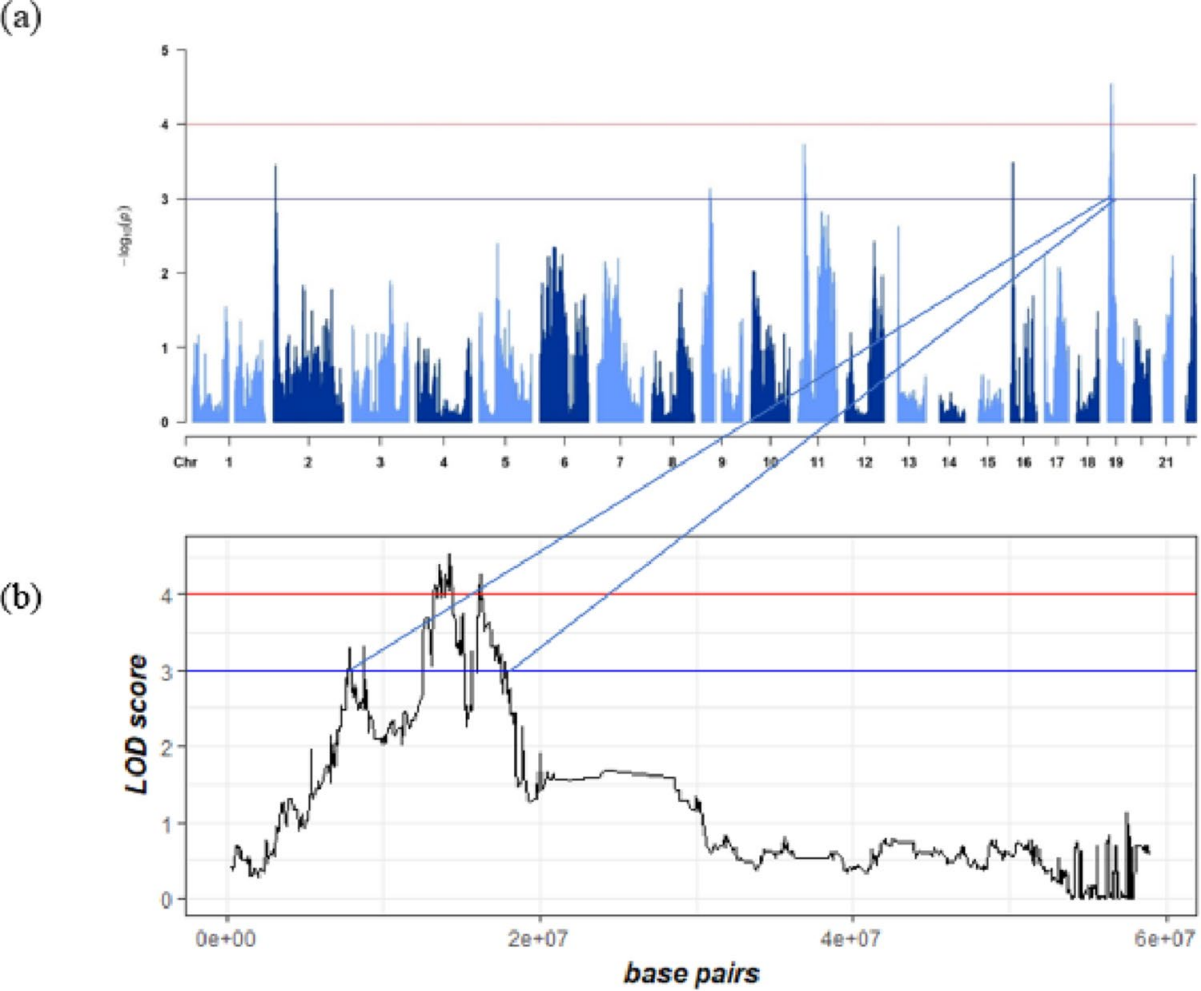
Plots of the **a** genome-wide linkage meta-analysis p-values (-log10(*P*)) of the NHGRI Family and NCR Family Cohorts and **b** for the significant region on chromosome 19 against the position of the variants where the horizontal blue lines correspond to LOD > 3.0 (~ *P* < 0.0001), while the horizontal red line corresponds to LOD > 4.0 (~ *P* < 0.00001). *DTI* diffusion tensor imaging, *fMRI* functional magnetic resonance imaging, *AF L* fractional anisotropy of left arcuate fasciculus, *AST L* fractional anisotropy of left anterior spinothalamic tract, *CC* fractional anisotropy of left corpus callosum, *IFOF R* fractional anisotropy of right inferior fronto-occipital fasciculus, *ILF* bilateral: fractional anisotropy of inferior longitudinal fasciculus, *SLF* bilateral: fractional anisotropy of superior longitudinal fasciculus, *UF L* fractional anisotropy of left uncinate fasciculus, Default mode—dorsal attention: connectivity between default mode and dorsal attention networks. Default mode -frontoparietal network: connectivity between default mode and frontoparietal control networks. Default mode—salience/ventral attention: connectivity between default mode and salience-ventral attention networks. Default mode—somatomotor network: connectivity between default mode and somatomotor network. P-values are shown only for nominal significant pairs (*p*< 0.05), with X indicating an FDR significant q-value (q < 0.05)

Two linkage regions on 2p25.2-p25.1 and 19p13.2-p13.11 reached a LOD score > 3.0 for the NHGRI Family Cohort (Figure S3), while 22q13.2-q13.31 reached the threshold for suggestive linkage (LOD > 2.0). For the NCR Family Cohort, while no significant linkage peaks were identified (at LOD > 3), there were suggestive linkage regions (at LOD > 2.0) at 3p26.3–26.2.2, 11p15.1 and 21q22.13–22.3.3 (Figure S4). For the meta-analysis, a linkage peak reaching the significant threshold LOD > 3.0 (*P* < 0.0001) was identified in a 4.9 Mb (± 1 Mb of chr19:13284603–16223287) region on 19p13.2–13.11.11 (Fig. 2), with five suggestive linkage peaks on 2p25.2-p25.1, 9p21-21.1, 11p15.1–14.2.2, 16p13.3-p13.2 and 22q13.2–13.31.31. We examined overlaps in these linkage signals with those reported in the prior literature (specifically 13 linkage regions with LOD > 3.0 in studies given in Table S3). We found that two of the six linkage regions (at LOD > 2.0) in our meta-analysis replicated previously reported linkage signals, with peaks on 2p25 and 16p13 (hypergeometric test *P* < 0.01).

### Overlap between linkage and FBAT signals

From the NHGRI Family Cohort, the 19p13.2–13.11.11 significant linkage peak overlaps with rs55741253, exonic to *DLL3* (*P* = 2.68 × 10^− 8^), while the meta-analysis suggestive linkage peak on 22q13.2-q13.31 encompasses rs1800206, exonic to *PPARA*, which had a *P* = 1.53 × 10^− 6^ in the FBAT meta-analysis.

### Comorbidity

Of the 430 children with ADHD in the NHGRI Family Cohort, 184 (42.8%) also had oppositional defiant disorder (ODD), and only 21 with ODD did not have ADHD. We found considerable similarity between the FBAT results for ADHD (regardless of the presence or absence of comorbid ODD) and those for ADHD with ODD, and ADHD without this comorbidity. Specifically, of the 19 genome-wide significant variants in the analysis of ADHD, five were genome-wide significant in the FBAT analysis of ADHD only. In the FBAT of ADHD with comorbid ODD, 16 of the 19 SNPs from the primary analysis retained genome-wide significance, while an additional 3 variants reached genome-wide significance- details in Table S4.

### Convergence between family-based analyses and GWAS findings

Using WebGestalt, we found that genes associated with ADHD in the NHGRI Family Cohort and in the meta-analysis enriched genes implicated in ADHD through GWAS (Demontis et al. 2023) (p-value = 0.013; FDR qvalue = 0.046). We also found that the polygenic risk score for ADHD, derived from independent data (Demontis et al. 2019), was associated with ADHD in both family cohorts. For the NCR Family Cohort, the maximal association was at the threshold of *P*_T_ =1 (Odds Ratio of 1.46, 95% confidence interval of 1.18 to 1.80, p-value = 0.0005; FDR qvalue = 0.0036) and for the NHGRI Family Cohort, the maximum association was at the threshold of *P*_T_ =0.1 (Odds Ratio of 1.18, 95% confidence interval of 1.05 to 1.34, p-value = 0.007; FDR qvalue = 0.07). Polygenic risk for autism spectrum disorder (ASD) was nominally associated (*P* < 0.05) with ADHD within the NCR Family Cohort only; otherwise, polygenic risk for other disorders was not tied to ADHD within either family cohort (Table S5).

### Gene based association and gene set enrichment analysis

Gene-based association analyses using MAGMA on the NHGRI Family Cohort analyses (Figure S5) identified three exome-wide significant genes (*CYP21A2*,* OR52I2* and *PTGER2; P* < 3.08 × 10^− 6^ (0.05/16192 genes). We submitted these three genes combined with the 31 genes implicated by an associated SNP GWAS signal in the NHGRI Family Cohort (*P* < 5 × 10^− 8^) to a gene-set enrichment analysis in the GENE2FUNC module in FUMA. There was significant enrichment of genes previously implicated by GWAS in autistic spectrum disorders or schizophrenia (Table 4). MAGMA did not identify any exome-wide genes associated with ADHD in the NCR cohort or the meta-analyses (Figure S6). Finally, we found enrichment by ADHD-associated genes in the NHGRI Family Cohort of two gene pathways: pattern recognition receptor activity (GO:0038187) and demethylase activity (GO:0032451) (Table S6).

**Table 4:** 34 genes for NHGRI family cohort: GWAS catalog reported genes for NHGRI family cohort

Gene set	*N*	*n*	*P*-value	FDR q-values	Genes
Autism spectrum disorder or schizophrenia	612	6	8.25E-05	3.53E-02	*PPP1R11*,* MUC22*,* C6orf15*,* PSORS1C1*,* CDSN*,* CYP21A2*
Hematology traits	25	3	3.83E-06	6.95E-03	*C6orf15*,* PSORS1C1*,* CDSN*
Lung cancer	187	4	7.83E-05	3.53E-02	*PITX2*,* MUC22*,* C6orf15*,* PSORS1C1*
White blood cell count	195	4	9.21E-05	3.53E-02	*PLCB1*,* C6orf15*,* PSORS1C1*,* CDSN*
Epstein-Barr virus immune response (EBNA-1)	12	2	9.73E-05	3.53E-02	*C6orf15*,* PSORS1C1*

*N* number of reported genes in gene set of GWAS catalog, *n* number of overlapped genes in our 34 genes with genes in gene set of GWAS catalog genes, *Genes* overlapped genes

### Overlap of ADHD genes and associated neural phenotypes

**Fig. 3:**
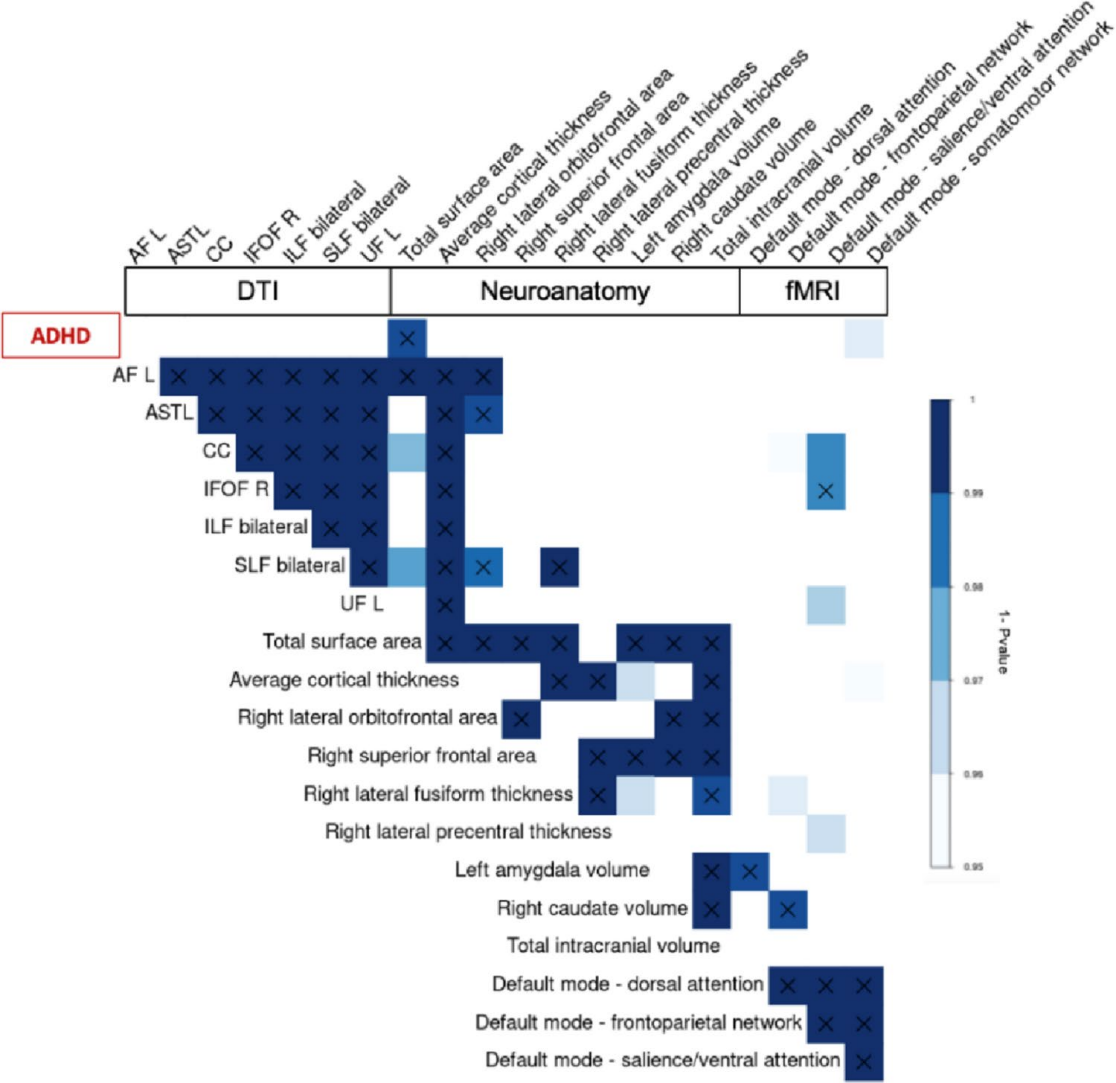
Gene overlap between ADHD with neuroimaging phenotypes for the NCR Family Cohort (*P* < 0.05)

For the NCR Family Cohort, we also identified genes associated with brain phenotypes, by first conducting FBAT analyses on these brain-based features. Using the GeneOverlap package, we found a significant overlapping between genes conferring ADHD risk within the NCR Family Cohort and genes associated with total white matter surface area (p-value: 0.006; q-value:0.019)- Fig. 3. There was also nominally significant genetic overlap between ADHD and one feature of the brain’s functional connectivity – connectivity between the default mode and somatomotor networks (p-value: 0.043).

## Discussion

There are four main findings in this report on families affected by ADHD. First, nineteen SNPs were associated with ADHD at genome-wide levels of significance in a cohort of 1538 individuals from 359 nuclear families. When combined meta-analytically with a smaller cohort of 157 families (631 individuals), three of these SNPs remained significant at genome-wide levels of significance and twelve were suggestive association (at *P* < 1 × 10^− 5^). Of these, only one (rs77176301) demonstrated a consistent direction of effect across both cohorts. This may be due to the small size of the NCR cohort and low allele frequencies, which can produce uninformative P_het_ scores. When comparing these findings to a recent ADHD GWAS (Demontis et al. 2023), no overlap at the SNP or gene level was observed with the loci identified in our family-based analyses. This suggests that the signals identified here may represent independent associations and may reflect differences in study design, where variants enriched in families may be too rare to detect in case–control association studies. We also searched for a replication between our gene sets and rare variants recently reported as associated with ADHD (Demontis et al. 2025). There was functional convergence at the pathway level, but no gene-family replication between the gene sets. For example, the KCNE2, KCNE3 and KCNA2 discovered in our study are neuronal/ion channel signaling genes, as are CACNA1D and ANK2 identified by Demontis et al. (2025).

Second, three regions segregating with ADHD in linkage analyses overlap significantly with previously identified ADHD linkage regions: 2p25.1 (LOD = 3.58) (Saviouk et al. 2011), 16p13.3-p13.2 (LOD = 4.2) (Smalley et al. 2002) and 19p13.2 (LOD = 1.72) (Arcos-Burgos et al. 2004). Third, we found a significant overlap between genes implicated in ADHD through our family-based analyses and those associated with ADHD in GWAS of unrelated cases and controls. Finally, using neuroimaging and genetic data obtained on the same individuals, we noted an overlap between the genes associated with ADHD and genes associated with some neuroanatomic and functional connectivity features.

The family-based analyses identify potential new candidate genes, which, if confirmed, may point to genes that are more strongly tied to familial than sporadic ADHD. The meta-analysis identified genome-wide significant associations implicating *TFRC*,* GSDME* and *TRIM31*. *TFRC* plays a role in ferroptosis, by importing iron into the cells from the extracellular environment (Feng et al. 2020). *GSDME* belongs to the gasdermin family of genes and plays a role in pryoptosis (De Schutter et al. 2021; Wang et al. 2017), and has been found to harbor variants identified as pleiotropic loci associated with multiple psychiatric disorders including ADHD (Cross-Disorder Group of the Psychiatric Genomics Consortium. Electronic address and Cross-Disorder Group of the Psychiatric Genomics 2019) *TRIM31* plays a role in the inflammatory response to infection (Rajsbaum, Garcia-Sastre, and Versteeg 2014) and was also found to be associated to intelligence in individuals ascertained for ADHD (Rizzi et al. 2011). The other gene emerging in the meta-analyses was *GSDME* (also known as *DFNA5*) which. Among the genes further implicated by the genome-wide significant associations in the larger NHGRI Family Cohort were *PEX2*, which has been found to have altered methylation at birth among those with childhood histories of ADHD (Walton et al. 2017); and *NF1B*, which was found to be haploinsufficient in 18 individuals with various neurodevelopmental phenotypes, including ADHD (Zhou et al. 2008; Schanze et al. 2018).

This study complements the recent GWAS using unrelated cases and controls from the PGC in two ways. First, we find that polygenic risk for ADHD, determined from the independent data of the PGC, was associated with ADHD within both family cohorts. This is perhaps unsurprising given that most of those with ADHD within the PGC cohorts are likely to have family histories of the disorder (Rovira et al. 2020). Second, the genes associated with familial ADHD in our study significantly enriched genes implicated in ADHD through the PGC GWAS. We note that polygenic risk for other psychiatric disorders did not associate with ADHD in the larger NHGRI Family Cohort, possibly reflecting its stringent exclusion criteria. There was however a nominally significant association between ADHD within the NCR Family Cohort and polygenic risk for ASD. In gene set enrichment of broader biological categories (using GO tools), there was enrichment of genes pertaining to demethylase activity, which might point to epigenetic dysregulation as a mechanism of interest in ADHD (Cecil and Nigg 2022). Further functional annotation of the identified loci, including integration of eQTL data from resources such as GTEx, may help refine gene prioritization and improve the biological interpretation of these findings in future studies.

We found an overlap between genes conferring risk for ADHD and those tied to variation in two neural features: total white surface area, and, at nominal levels of significance, a feature of functional connectivity feature that reflected interactions between the default mode network and a network involved in motor planning and action. The latter finding is of note as an influential model of ADHD posits the disorder to stem partly from dysregulated connectivity between the ‘task-oriented’ networks that support motor control and the default-mode network that is prominent during task-free, introspective processing (Sonuga-Barke and Castellanos 2007; Sutcubasi et al. 2020; Sudre et al. 2017, 2018). An analogy is that the online activity of the brain in motor planning and execution is repeatedly interrupted by the brain going ‘offline’, leading to symptoms characteristics of impulsivity and hyperactivity (Duffy et al. 2021, Rosenberg et al. 2016, Bozhilova et al. 2018).

There are several limitations. Firstly, we consider only genotype array data and thus lacked the whole exome or whole genome sequences that are needed to capture rare coding and noncoding variants and some forms of structural variation that may be associated with ADHD. The cohorts were genotyped on different arrays, with less than 100,000 shared variants available for analysis prior to imputation. We limited the analysis of imputed data to variants with a MAF > 5%, since variants with lower frequencies have higher imputation inaccuracies, especially when dealing with family data (Stahl et al. 2021). While we were able to show similarity of associations for those who had ADHD either with or without comorbid ODD, other comorbid disorders were not prevalent enough to allow for a systematic exploration of their genetic associations within our families. Our cohorts also lacked detailed information across both cohorts regarding exposures to environmental factors, such as childhood adversity, thus not allowing to test for the possibility of a gene-environment correlation with our candidate genes.

In conclusion, using a family-based approach, we report some novel genomic associations and linkage regions with familial ADHD and note overlaps with recent GWAS studies. In preliminary work, we point to changes in brain surface area and functional connectivity as candidates for the neural substrates underpinning these genomic findings.

## Supplementary Information

Below is the link to the electronic supplementary material.

Supplementary Material 1

## Data Availability

No datasets were generated or analysed during the current study. Data are being consented from the NHGRI Family Cohort for dbGaP (pending final approvals and accession number). Data from the NCR Family Cohort will be deposited in dbGaP on all who gave consent for sharing (accession number pending).

## Code availability

No custom code was developed for this study.

## References

[CR1] Abecasis GR, Cherny SS, Cookson WO, Cardon LR (2002) Merlin–rapid analysis of dense genetic maps using sparse gene flow trees. Nat Genet 30:97–101 11731797 10.1038/ng786

[CR2] Acosta MT, Castellanos FX, Bolton KL, Balog JZ, Eagen P, Nee L, Jones J, Palacio L, Sarampote C, Russell HF, Berg K, Arcos-Burgos M, Muenke M (2008) Latent class subtyping of attention-deficit/hyperactivity disorder and comorbid conditions. J Am Acad Child Adolesc Psychiatry 47:797–807 18520958 10.1097/CHI.0b013e318173f70b PMC2774844

[CR3] Anney RJ, Lasky-Su J, O’Dúshláine C, Kenny E, Neale BM, Mulligan A, Franke B, Zhou K, Chen W, Christiansen H, Arias-Vásquez A, Banaschewski T, Buitelaar J, Ebstein R, Miranda A, Mulas F, Oades RD, Roeyers H, Rothenberger A, Sergeant J, Sonuga-Barke E, Steinhausen H, Asherson P, Faraone SV, Gill M (2008) Conduct disorder and ADHD: evaluation of conduct problems as a categorical and quantitative trait in the international multicentre ADHD genetics study. Am J Med Genet B Neuropsychiatr Genet 147B(8):1369–78. 10.1002/ajmg.b.30871 18951430 10.1002/ajmg.b.30871

[CR4] Arcos-Burgos M, Castellanos FX, Pineda D, Lopera F, Palacio JD, Palacio LG, Rapoport JL, Berg K, Bailey-Wilson JE, Muenke M (2004) Attention-deficit/hyperactivity disorder in a population isolate: linkage to loci at 4q13.2, 5q33.3, 11q22, and 17p11. Am J Hum Genet 75:998–1014 15497111 10.1086/426154 PMC1182160

[CR5] Asherson P, Zhou K, Anney RJ, Franke B, Buitelaar J, Ebstein R, Gill M, Altink M, Arnold R, Boer F, Brookes K, Buschgens C, Butler L, Cambell D, Chen W, Christiansen H, Feldman L, Fleischman K, Fliers E, Howe-Forbes R, Goldfarb A, Heise A, Gabriels I, Johansson L, Lubetzki I, Marco R, Medad S, Minderaa R, Mulas F, Muller U, Mulligan A, Neale B, Rijsdijk F, Rabin K, Rommelse N, Sethna V, Sorohan J, Uebel H, Psychogiou L, Weeks A, Barrett R, Xu X, Banaschewski T, Sonuga-Barke E, Eisenberg J, Manor I, Miranda A, Oades RD, Roeyers H, Rothenberger A, Sergeant J, Steinhausen HC, Taylor E, Thompson M, Faraone SV (2008) A high-density SNP linkage scan with 142 combined subtype ADHD sib pairs identifies linkage regions on chromosomes 9 and 16 Mol Psychiatry 13:514−21 10.1038/sj.mp.4002140 18180756

[CR6] Bozhilova NS, Michelini G, Kuntsi J, Asherson P (2018) Mind wandering perspective on attention-deficit/hyperactivity disorder. Neurosci Biobehav Rev. 92:464–476 Epub 2018 Jul 20. PMID: 30036553; PMCID: PMC6525148. 10.1016/j.neubiorev.2018.07.010 10.1016/j.neubiorev.2018.07.010 PMC6525148 30036553

[CR7] Cecil CAM, Nigg JT (2022) Epigenetics and ADHD: reflections on current knowledge, research priorities and translational potential. Mol Diagn Ther 26:581–606 35933504 10.1007/s40291-022-00609-y PMC7613776

[CR8] Chen H, Wang C, Conomos MP, Stilp AM, Li Z, Sofer T, Szpiro AA, Chen W, Brehm JM, Celedon JC, Redline S, Papanicolaou GJ, Thornton TA, Laurie CC, Rice K, Lin X (2016) Control for population structure and relatedness for binary traits in genetic association studies via logistic mixed models. Am J Hum Genet 98:653–66 27018471 10.1016/j.ajhg.2016.02.012 PMC4833218

[CR9] Choi SW, O’Reilly PF (2019) PRSice-2: Polygenic Risk Score software for biobank-scale data. Gigascience 8 10.1093/gigascience/giz082 PMC6629542 31307061

[CR10] Conners KC, MHS Staff (2004) Conners’ Continuous Performance Test II. CPT II. Multi-Health Systems, North Tonawanda, NY

[CR11] Conomos MP, Miller MB, Thornton TA (2015) Robust inference of population structure for ancestry prediction and correction of stratification in the presence of relatedness. Genet Epidemiol 39:276−93 10.1002/gepi.21896 PMC4836868 25810074

[CR12] Cross-Disorder Group of the Psychiatric Genomics Consortium. Electronic address, plee mgh harvard edu, and Consortium Cross-Disorder Group of the Psychiatric Genomics. (2019) Genomic Relationships, Novel Loci, and Pleiotropic Mechanisms across Eight Psychiatric Disorders. Cell. 179:1469−82 e11 10.1016/j.cell.2019.11.020 PMC7077032 31835028

[CR13] Das S, Forer L, Schonherr S, Sidore C, Locke AE, Kwong A, Vrieze SI, Chew EY, Levy S, McGue M, Schlessinger D, Stambolian D, Loh PR, Iacono WG, Swaroop A, Scott LJ, Cucca F, Kronenberg F, Boehnke M, Abecasis GR, Fuchsberger C (2016) Next-generation genotype imputation service and methods. Nat Genet. 48:1284-87 10.1038/ng.3656 PMC5157836 27571263

[CR14] de Leeuw CA, Mooij JM, Heskes T, Posthuma D (2015) MAGMA: generalized gene-set analysis of GWAS data. PLoS Comput Biol 11:e1004219 10.1371/journal.pcbi.1004219 PMC4401657 25885710

[CR15] De Schutter E, Ramon J, Pfeuty B, De Tender C, Stremersch S, Raemdonck K, de Beeck KO, Declercq W, Riquet FB, Braeckmans K, Vandenabeele P (2021) Plasma membrane perforation by GSDME during apoptosis-driven secondary necrosis. Cell Mol Life Sci. 79:19 10.1007/s00018-021-04078-0 PMC8720079 34971436

[CR16] Demontis, D, R. K. Walters, J. Martin, M. Mattheisen, T. D. Als, E. Agerbo, G. Baldursson, R. Belliveau, J. Bybjerg-Grauholm, M. Baekvad-Hansen, F. Cerrato, K. Chambert, C. Churchhouse, A. Dumont, N. Eriksson, M. Gandal, J. I. Goldstein, K. L. Grasby, J. Grove, O. O. Gudmundsson, C. S. Hansen, M. E. Hauberg, M. V. Hollegaard, D. P. Howrigan, H. Huang, J. B. Maller, A. R. Martin, N. G. Martin, J. Moran, J. Pallesen, D. S. Palmer, C. B. Pedersen, M. G. Pedersen, T. Poterba, J. B. Poulsen, S. Ripke, E. B. Robinson, F. K. Satterstrom, H. Stefansson, C. Stevens, P. Turley, G. B. Walters, H. Won, M. J. Wright, Adhd Working Group of the Psychiatric Genomics Consortium, Lifecourse Early, Consortium Genetic Epidemiology, Team andMe Research, O. A. Andreassen, P. Asherson, C. L. Burton, D. I. Boomsma, B. Cormand, S. Dalsgaard, B. Franke, J. Gelernter, D. Geschwind, H. Hakonarson, J. Haavik, H. R. Kranzler, J. Kuntsi, K. Langley, K. P. Lesch, C. Middeldorp, A. Reif, L. A. Rohde, P. Roussos, R. Schachar, P. Sklar, E. J. S. Sonuga-Barke, P. F. Sullivan, A. Thapar, J. Y. Tung, I. D. Waldman, S. E. Medland, K. Stefansson, M. Nordentoft, D. M. Hougaard, T. Werge, O. Mors, P. B. Mortensen, M. J. Daly, S. V. Faraone, A. D. Borglum, and B. M. Neale (2019) Discovery of the first genome-wide significant risk loci for attention deficit/hyperactivity disorder. Nat Genet 51:63–75 10.1038/s41588-018-0269-7 PMC6481311 30478444

[CR17] Demontis, Ditte, G. Bragi Walters, Georgios Athanasiadis, Raymond Walters, Karen Therrien, Trine Tollerup Nielsen, Leila Farajzadeh, Georgios Voloudakis, Jaroslav Bendl, Biau Zeng, Wen Zhang, Jakob Grove, Thomas D. Als, Jinjie Duan, F. Kyle Satterstrom, Jonas Bybjerg-Grauholm, Marie Bækved-Hansen, Olafur O. Gudmundsson, Sigurdur H. Magnusson, Gisli Baldursson, Katrin Davidsdottir, Gyda S. Haraldsdottir, Esben Agerbo, Gabriel E. Hoffman, Søren Dalsgaard, Joanna Martin, Marta Ribasés, Dorret I. Boomsma, Maria Soler Artigas, Nina Roth Mota, Daniel Howrigan, Sarah E. Medland, Tetyana Zayats, Veera M. Rajagopal, Alexandra Havdahl, Alysa Doyle, Andreas Reif, Anita Thapar, Bru Cormand, Calwing Liao, Christie Burton, Claiton H. D. Bau, Diego Luiz Rovaris, Edmund Sonuga-Barke, Elizabeth Corfield, Eugenio Horacio Grevet, Henrik Larsson, Ian R. Gizer, Irwin Waldman, Isabell Brikell, Jan Haavik, Jennifer Crosbie, James McGough, Joanna Kuntsi, Joseph Glessner, Kate Langley, Klaus-Peter Lesch, Luis Augusto Rohde, Mara H. Hutz, Marieke Klein, Mark Bellgrove, Martin Tesli, Michael C. O’Donovan, Ole Andreas Andreassen, Patrick W. L. Leung, Pedro M. Pan, Ridha Joober, Russel Schachar, Sandra Loo, Stephanie H. Witt, Ted Reichborn-Kjennerud, Tobias Banaschewski, Ziarih Hawi, Mark J. Daly, Ole Mors, Merete Nordentoft, Ole Mors, David M. Hougaard, Preben Bo Mortensen, Mark J. Daly, Stephen V. Faraone, Hreinn Stefansson, Panos Roussos, Barbara Franke, Thomas Werge, Benjamin M. Neale, Kari Stefansson, Anders D. Børglum, Adhd Working Group of the Psychiatric Genomics Consortium, and Psych-Broad Consortium i. (2023) Genome-wide analyses of ADHD identify 27 risk loci, refine the genetic architecture and implicate several cognitive domains. Nature Genetics 10.1038/s41588-022-01285-8 PMC10914347 36702997

[CR18] Demontis D, Duan J, Hsu YH, Pintacuda G, Grove J, Nielsen TT, Thirstrup J, Martorana M, Botts T, Satterstrom FK, Bybjerg-Grauholm J, Tsai JHY, Glerup S, Hoogman M, Buitelaar J, Klein M, Ziegler GC, Jacob C, Grimm O, Bayas M, Kobayashi NF, Kittel-Schneider S, Lesch KP, Franke B, Reif A, Agerbo E, Werge T, Nordentoft M, Mors O, Mortensen PB, Lage K, Daly MJ, Neale BM, Børglum AD (2026) Rare genetic variants confer a high risk of ADHD and implicate neuronal biology. Nature. 649(8098):909–917 10.1038/s41586-025-09702-8 PMC12823435 41224997

[CR19] Duffy Kelly A, Keri S Rosch, Mary Beth Nebel, Karen E Seymour, Martin A Lindquist, James J Pekar, Stewart H Mostofsky, Jessica R Cohen (2021) Increased integration between default mode and task-relevant networks in children with ADHD is associated with impaired response control. Dev Cogn Neurosci 50:100980 10.1016/j.dcn.2021.100980 PMC8278154 34252881

[CR20] Epstein J, Diane E. Johnson, Conners CK (2001) ‘Conners’ Adult ADHD Diagnostic Interview for DSM-IV™’.

[CR21] Faraone SV, Larsson H (2019) Genetics of attention deficit hyperactivity disorder. Mol Psychiatry. 24:562−75 10.1038/s41380-018-0070-0 PMC6477889 29892054

[CR22] Feng H, Schorpp K, Jin J, Yozwiak CE, Hoffstrom BG, Decker AM, Rajbhandari P, Stokes ME, Bender HG, Csuka JM, Upadhyayula PS, Canoll P, Uchida K, Soni RK, Hadian K, Stockwell BR (2020) Transferrin Receptor Is a Specific Ferroptosis Marker. Cell Rep. 30:3411−23 e7 10.1016/j.celrep.2020.02.049 PMC7172030 32160546

[CR23] First, Michael B, Robert L Spitzer, Miriam Gibbon, and Janet BW Williams (2002) “Structured clinical interview for DSM-IV-TR axis I disorders, research version, patient edition. SCID-I/P New York/

[CR24] Grove, J, S. Ripke, T. D. Als, M. Mattheisen, R. K. Walters, H. Won, J. Pallesen, E. Agerbo, O. A. Andreassen, R. Anney, S. Awashti, R. Belliveau, F. Bettella, J. D. Buxbaum, J. Bybjerg-Grauholm, M. Baekvad-Hansen, F. Cerrato, K. Chambert, J. H. Christensen, C. Churchhouse, K. Dellenvall, D. Demontis, S. De Rubeis, B. Devlin, S. Djurovic, A. L. Dumont, J. I. Goldstein, C. S. Hansen, M. E. Hauberg, M. V. Hollegaard, S. Hope, D. P. Howrigan, H. Huang, C. M. Hultman, L. Klei, J. Maller, J. Martin, A. R. Martin, J. L. Moran, M. Nyegaard, T. Naerland, D. S. Palmer, A. Palotie, C. B. Pedersen, M. G. Pedersen, T. dPoterba, J. B. Poulsen, B. S. Pourcain, P. Qvist, K. Rehnstrom, A. Reichenberg, J. Reichert, E. B. Robinson, K. Roeder, P. Roussos, E. Saemundsen, S. Sandin, F. K. Satterstrom, G. Davey Smith, H. Stefansson, S. Steinberg, C. R. Stevens, P. F. Sullivan, P. Turley, G. B. Walters, X. Xu, Consortium Autism Spectrum Disorder Working Group of the Psychiatric Genomics, Bupgen, Consortium Major Depressive Disorder Working Group of the Psychiatric Genomics, Team andMe Research, K. Stefansson, D. H. Geschwind, M. Nordentoft, D. M. Hougaard, T. Werge, O. Mors, P. B. Mortensen, B. M. Neale, M. J. Daly, and A. D. Borglum (2019) Identification of common genetic risk variants for autism spectrum disorder. Nat Genet 51:431−44 10.1038/s41588-019-0344-8 PMC6454898 30804558

[CR25] Hebebrand J, Dempfle A, Saar K, Thiele H, Herpertz-Dahlmann B, Linder M, Kiefl H, Remschmidt H, Hemminger U, Warnke A, Knolker U, Heiser P, Friedel S, Hinney A, Schafer H, Nurnberg P, Konrad K (2006) A genome-wide scan for attention-deficit/hyperactivity disorder in 155 German sib-pairs. Mol Psychiatry 11:196–205 10.1038/sj.mp.4001761 16222334

[CR26] Hoogman M, Buitelaar JK, Faraone SV, Shaw P, Franke B, Enigma-Adhd working group. (2017) Subcortical brain volume differences in participants with attention deficit hyperactivity disorder in children and adults - Authors’ reply. Lancet Psychiatry 4:440−41 10.1016/S2215-0366(17)30200-6 28495548

[CR27] Hoogman, M., R. Muetzel, J. P. Guimaraes, E. Shumskaya, M. Mennes, M. P. Zwiers, N. Jahanshad, G. Sudre, T. Wolfers, E. A. Earl, J. C. Soliva Vila, Y. Vives-Gilabert, S. Khadka, S. E. Novotny, C. A. Hartman, D. J. Heslenfeld, L. J. S. Schweren, S. Ambrosino, B. Oranje, P. de Zeeuw, T. M. Chaim-Avancini, P. G. P. Rosa, M. V. Zanetti, C. B. Malpas, G. Kohls, G. G. von Polier, J. Seitz, J. Biederman, A. E. Doyle, A. M. Dale, T. G. M. van Erp, J. N. Epstein, T. L. Jernigan, R. Baur-Streubel, G. C. Ziegler, K. C. Zierhut, A. Schrantee, M. F. Hovik, A. J. Lundervold, C. Kelly, H. McCarthy, N. Skokauskas, R. L. O’Gorman Tuura, A. Calvo, S. Lera-Miguel, R. Nicolau, K. C. Chantiluke, A. Christakou, A. Vance, M. Cercignani, M. C. Gabel, P. Asherson, S. Baumeister, D. Brandeis, S. Hohmann, I. E. Bramati, F. Tovar-Moll, A. J. Fallgatter, B. Kardatzki, L. Schwarz, A. Anikin, A. Baranov, T. Gogberashvili, D. Kapilushniy, A. Solovieva, H. El Marroun, T. White, G. Karkashadze, L. Namazova-Baranova, T. Ethofer, P. Mattos, T. Banaschewski, D. Coghill, K. J. Plessen, J. Kuntsi, M. A. Mehta, Y. Paloyelis, N. A. Harrison, M. A. Bellgrove, T. J. Silk, A. I. Cubillo, K. Rubia, L. Lazaro, S. Brem, S. Walitza, T. Frodl, M. Zentis, F. X. Castellanos, Y. N. Yoncheva, J. Haavik, L. Reneman, A. Conzelmann, K. P. Lesch, P. Pauli, A. Reif, L. Tamm, K. Konrad, E. Oberwelland Weiss, G. F. Busatto, M. R. Louza, S. Durston, P. J. Hoekstra, J. Oosterlaan, M. C. Stevens, J. A. Ramos-Quiroga, O. Vilarroya, D. A. Fair, J. T. Nigg, P. M. Thompson, J. K. Buitelaar, S. V. Faraone, P. Shaw, H. Tiemeier, J. Bralten, and B. Franke. 2019. ‘Brain Imaging of the Cortex in ADHD: A Coordinated Analysis of Large-Scale Clinical and Population-Based Samples’, *Am J Psychiatry*, 176: 531 − 42. 10.1176/appi.ajp.2019.18091033 PMC6879185 31014101

[CR28] International Obsessive Compulsive Disorder Foundation Genetics, Collaborative, and O. C. D. Collaborative Genetics Association Studies (2018) Revealing the complex genetic architecture of obsessive-compulsive disorder using meta-analysis. Mol Psychiatry 23:1181−88 10.1038/mp.2017.154 PMC6660151 28761083

[CR29] Kong A, Cox NJ (1997) Allele-sharing models: LOD scores and accurate linkage tests. Am J Hum Genet 61:1179−88 10.1086/301592 PMC1716027 9345087

[CR30] Laird NM, Horvath S, Xu X (2000) Implementing a unified approach to family-based tests of association. Genet Epidemiol 19 Suppl 1:S36−42 10.1002/1098-2272(2000)19:1+<::AID-GEPI6>3.0.CO;2-M 11055368

[CR31] Lander E, Kruglyak L (1995) Genetic dissection of complex traits: guidelines for interpreting and reporting linkage results. Nat Genet 11(3):241−7 10.1038/ng1195-241 7581446

[CR32] Lange EM, Sun J, Lange LA, Zheng SL, Duggan D, Carpten JD, Gronberg H, Isaacs WB, Xu J, Chang BL (2008) Family-based samples can play an important role in genetic association studies. Cancer Epidemiol Biomarkers Prev 17:2208−14 10.1158/1055-9965.EPI-08-0183 PMC2665689 18768484

[CR33] Mullins N, Forstner AJ, O'Connell KS, Coombes B, Coleman JRI, Qiao Z, Als TD, Bigdeli TB, Børte S, Bryois J, Charney AW, Drange OK, Gandal MJ, Hagenaars SP, Ikeda M, Kamitaki N, Kim M, Krebs K, Panagiotaropoulou G, Schilder BM, Sloofman LG, Steinberg S, Trubetskoy V, Winsvold BS, Won HH, Abramova L, Adorjan K, Agerbo E, Al Eissa M, Albani D, Alliey-Rodriguez N, Anjorin A, Antilla V, Antoniou A, Awasthi S, Baek JH, Bækvad-Hansen M, Bass N, Bauer M, Beins EC, Bergen SE, Birner A, Bøcker Pedersen C, Bøen E, Boks MP, Bosch R, Brum M, Brumpton BM, Brunkhorst-Kanaan N, Budde M, Bybjerg-Grauholm J, Byerley W, Cairns M, Casas M, Cervantes P, Clarke TK, Cruceanu C, Cuellar-Barboza A, Cunningham J, Curtis D, Czerski PM, Dale AM, Dalkner N, David FS, Degenhardt F, Djurovic S, Dobbyn AL, Douzenis A, Elvsåshagen T, Escott-Price V, Ferrier IN, Fiorentino A, Foroud TM, Forty L, Frank J, Frei O, Freimer NB, Frisén L, Gade K, Garnham J, Gelernter J, Giørtz Pedersen M, Gizer IR, Gordon SD, Gordon-Smith K, Greenwood TA, Grove J, Guzman-Parra J, Ha K, Haraldsson M, Hautzinger M, Heilbronner U, Hellgren D, Herms S, Hoffmann P, Holmans PA, Huckins L, Jamain S, Johnson JS, Kalman JL, Kamatani Y, Kennedy JL, Kittel-Schneider S, Knowles JA, Kogevinas M, Koromina M, Kranz TM, Kranzler HR, Kubo M, Kupka R, Kushner SA, Lavebratt C, Lawrence J, Leber M, Lee HJ, Lee PH, Levy SE, Lewis C, Liao C, Lucae S, Lundberg M, MacIntyre DJ, Magnusson SH, Maier W, Maihofer A, Malaspina D, Maratou E, Martinsson L, Mattheisen M, McCarroll SA, McGregor NW, McGuffin P, McKay JD, Medeiros H, Medland SE, Millischer V, Montgomery GW, Moran JL, Morris DW, Mühleisen TW, O'Brien N, O'Donovan C, Olde Loohuis LM, Oruc L, Papiol S, Pardiñas AF, Perry A, Pfennig A, Porichi E, Potash JB, Quested D, Raj T, Rapaport MH, DePaulo JR, Regeer EJ, Rice JP, Rivas F, Rivera M, Roth J, Roussos P, Ruderfer DM, Sánchez-Mora C, Schulte EC, Senner F, Sharp S, Shilling PD, Sigurdsson E, Sirignano L, Slaney C, Smeland OB, Smith DJ, Sobell JL, Søholm Hansen C, Soler Artigas M, Spijker AT, Stein DJ, Strauss JS, Świątkowska B, Terao C, Thorgeirsson TE, Toma C, Tooney P, Tsermpini EE, Vawter MP, Vedder H, Walters JTR, Witt SH, Xi S, Xu W, Yang JMK, Young AH, Young H, Zandi PP, Zhou H, Zillich L; HUNT All-In Psychiatry; Adolfsson R, Agartz I, Alda M, Alfredsson L, Babadjanova G, Backlund L, Baune BT, Bellivier F, Bengesser S, Berrettini WH, Blackwood DHR, Boehnke M, Børglum AD, Breen G, Carr VJ, Catts S, Corvin A, Craddock N, Dannlowski U, Dikeos D, Esko T, Etain B, Ferentinos P, Frye M, Fullerton JM, Gawlik M, Gershon ES, Goes FS, Green MJ, Grigoroiu-Serbanescu M, Hauser J, Henskens F, Hillert J, Hong KS, Hougaard DM, Hultman CM, Hveem K, Iwata N, Jablensky AV, Jones I, Jones LA, Kahn RS, Kelsoe JR, Kirov G, Landén M, Leboyer M, Lewis CM, Li QS, Lissowska J, Lochner C, Loughland C, Martin NG, Mathews CA, Mayoral F, McElroy SL, McIntosh AM, McMahon FJ, Melle I, Michie P, Milani L, Mitchell PB, Morken G, Mors O, Mortensen PB, Mowry B, Müller-Myhsok B, Myers RM, Neale BM, Nievergelt CM, Nordentoft M, Nöthen MM, O'Donovan MC, Oedegaard KJ, Olsson T, Owen MJ, Paciga SA, Pantelis C, Pato C, Pato MT, Patrinos GP, Perlis RH, Posthuma D, Ramos-Quiroga JA, Reif A, Reininghaus EZ, Ribasés M, Rietschel M, Ripke S, Rouleau GA, Saito T, Schall U, Schalling M, Schofield PR, Schulze TG, Scott LJ, Scott RJ, Serretti A, Shannon Weickert C, Smoller JW, Stefansson H, Stefansson K, Stordal E, Streit F, Sullivan PF, Turecki G, Vaaler AE, Vieta E, Vincent JB, Waldman ID, Weickert TW, Werge T, Wray NR, Zwart JA, Biernacka JM, Nurnberger JI, Cichon S, Edenberg HJ, Stahl EA, McQuillin A, Di Florio A, Ophoff RA, Andreassen OA (2021) Genome-wide association study of more than 40,000 bipolar disorder cases provides new insights into the underlying biology. Nat Genet. 53(6):817–829. Epub 2021 May 17. PMID: 34002096; PMCID: PMC8192451 10.1038/s41588-021-00857-4 10.1038/s41588-021-00857-4 PMC8192451 34002096

[CR34] Neale, B. M., S. E. Medland, S. Ripke, P. Asherson, B. Franke, K. P. Lesch, S. V. Faraone, T. T. Nguyen, H. Schafer, P. Holmans, M. Daly, H. C. Steinhausen, C. Freitag, A. Reif, T. J. Renner, M. Romanos, J. Romanos, S. Walitza, A. Warnke, J. Meyer, H. Palmason, J. Buitelaar, A. A. Vasquez, N. Lambregts-Rommelse, M. Gill, R. J. Anney, K. Langely, M. O’Donovan, N. Williams, M. Owen, A. Thapar, L. Kent, J. Sergeant, H. Roeyers, E. Mick, J. Biederman, A. Doyle, S. Smalley, S. Loo, H. Hakonarson, J. Elia, A. Todorov, A. Miranda, F. Mulas, R. P. Ebstein, A. Rothenberger, T. Banaschewski, R. D. Oades, E. Sonuga-Barke, J. McGough, L. Nisenbaum, F. Middleton, X. Hu, S. Nelson, and Gwas Consortium Adhd Subgroup Psychiatric (2010) Meta-analysis of genome-wide association studies of attention-deficit/hyperactivity disorder. J Am Acad Child Adolesc Psychiatry 49:884−97 10.1016/j.jaac.2010.06.008 PMC2928252 20732625

[CR35] Norman, Luke J, Gustavo Sudre, Jolie Price, Gauri G Shastri, and Philip Shaw (2022) Evidence from “big data” for the default-mode hypothesis of ADHD: a mega-analysis of multiple large samples. Neuropsychopharmacology 1–9 10.1038/s41386-022-01408-z PMC9751118 36100657

[CR36] Oades RD, Lasky-Su J, Christiansen H, Faraone SV, Sonuga-Barke EJ, Banaschewski T, Chen W, Anney RJ, Buitelaar JK, Ebstein RP, Franke B, Gill M, Miranda A, Roeyers H, Rothenberger A, Sergeant JA, Steinhausen HC, Taylor EA, Thompson M, Asherson P. The influence of serotonin- and other genes on impulsive behavioral aggression and cognitive impulsivity in children with attention-deficit/hyperactivity disorder (ADHD): Findings from a family-based association test (FBAT) analysis. Behav Brain Funct. 2008 Oct 20;4:48. 10.1186/1744-9081-4-48 PMC2577091 18937842

[CR37] Ogdie MN, Fisher SE, Yang M, Ishii J, Francks C, Loo SK, Cantor RM, McCracken JT, McGough JJ, Smalley SL, Nelson SF (2004) Attention deficit hyperactivity disorder: fine mapping supports linkage to 5p13, 6q12, 16p13, and 17p11. Am J Hum Genet 75:661−8 10.1086/424387 PMC1182053 15297934

[CR38] Pardinas, A. F., P. Holmans, A. J. Pocklington, V. Escott-Price, S. Ripke, N. Carrera, S. E. Legge, S. Bishop, D. Cameron, M. L. Hamshere, J. Han, L. Hubbard, A. Lynham, K. Mantripragada, E. Rees, J. H. MacCabe, S. A. McCarroll, B. T. Baune, G. Breen, E. M. Byrne, U. Dannlowski, T. C. Eley, C. Hayward, N. G. Martin, A. M. McIntosh, R. Plomin, D. J. Porteous, N. R. Wray, A. Caballero, D. H. Geschwind, L. M. Huckins, D. M. Ruderfer, E. Santiago, P. Sklar, E. A. Stahl, H. Won, E. Agerbo, T. D. Als, O. A. Andreassen, M. Baekvad-Hansen, P. B. Mortensen, C. B. Pedersen, A. D. Borglum, J. Bybjerg-Grauholm, S. Djurovic, N. Durmishi, M. G. Pedersen, V. Golimbet, J. Grove, D. M. Hougaard, M. Mattheisen, E. Molden, O. Mors, M. Nordentoft, M. Pejovic-Milovancevic, E. Sigurdsson, T. Silagadze, C. S. Hansen, K. Stefansson, H. Stefansson, S. Steinberg, S. Tosato, T. Werge, Gerad Consortium, Crestar Consortium, D. A. Collier, D. Rujescu, G. Kirov, M. J. Owen, M. C. O’Donovan, and J. T. R. Walters (2018) Common schizophrenia alleles are enriched in mutation-intolerant genes and in regions under strong background selection. Nat Genet. 50:381−89 10.1038/s41588-018-0059-2 PMC5918692 29483656

[CR39] Purcell S, Neale B, Todd-Brown K, Thomas L, Ferreira MA, Bender D, Maller J, Sklar P, de Bakker PI, Daly MJ, Sham PC (2007) PLINK: a tool set for whole-genome association and population-based linkage analyses. Am J Hum Genet 81:559–75 10.1086/519795 PMC1950838 17701901

[CR40] Rabinowitz D, Laird N (2000) A unified approach to adjusting association tests for population admixture with arbitrary pedigree structure and arbitrary missing marker information. Hum Hered 50:211–23 10782012 10.1159/000022918

[CR41] Rajsbaum R, Garcia-Sastre A, Versteeg GA (2014) TRIMmunity: the roles of the TRIM E3-ubiquitin ligase family in innate antiviral immunity. J Mol Biol 426:1265–84 24333484 10.1016/j.jmb.2013.12.005 PMC3945521

[CR42] Reich W (2000) Diagnostic interview for children and adolescents (DICA). J Am Acad Child Adolesc Psychiatry 39:59–66 10638068 10.1097/00004583-200001000-00017

[CR43] Rizzi TS, Arias-Vasquez A, Rommelse N, Kuntsi J, Anney R, Asherson P, Buitelaar J, Banaschewski T, Ebstein R, Ruano D, Van der Sluis S, Markunas CA, Garrett ME, Ashley-Koch AE, Kollins SH, Anastopoulos AD, Hansell NK, Wright MJ, Montgomery GW, Martin NG, Harris SE, Davies G, Tenesa A, Porteous DJ, Starr JM, Deary IJ, St Pourcain B, Davey Smith G, Timpson NJ, Evans DM, Gill M, Miranda A, Mulas F, Oades RD, Roeyers H, Rothenberger A, Sergeant J, Sonuga-Barke E, Steinhausen HC, Taylor E, Faraone SV, Franke B, Posthuma D (2011) The ATXN1 and TRIM31 genes are related to intelligence in an ADHD background: evidence from a large collaborative study totaling 4,963 subjects. Am J Med Genet B Neuropsychiatr Genet 156:145–57 10.1002/ajmg.b.31149 PMC3085124 21302343

[CR44] Romanos M, Freitag C, Jacob C, Craig DW, Dempfle A, Nguyen TT, Halperin R, Walitza S, Renner TJ, Seitz C, Romanos J, Palmason H, Reif A, Heine M, Windemuth-Kieselbach C, Vogler C, Sigmund J, Warnke A, Schafer H, Meyer J, Stephan DA, Lesch KP (2008) Genome-wide linkage analysis of ADHD using high-density SNP arrays: novel loci at 5q13.1 and 14q12. Mol Psychiatry 13:522–30 18301393 10.1038/mp.2008.12

[CR45] Rosenberg MD, Finn ES, Scheinost D, Papademetris X, Shen X, Constable RT, Chun MM (2016) A neuromarker of sustained attention from whole-brain functional connectivity. Nat Neurosci 19:165 26595653 10.1038/nn.4179 PMC4696892

[CR46] Rosenberg, Leon E., and Diane Drobnis Rosenberg (2012) Human genes and genomes : science, health, society. Elsevier Academic Press: London ; Waltham.

[CR47] Rovira P, Demontis D, Sanchez-Mora C, Zayats T, Klein M, Mota NR, Weber H, Garcia-Martinez I, Pagerols M, Vilar-Ribo L, Arribas L, Richarte V, Corrales M, Fadeuilhe C, Bosch R, Martin GE, Almos P, Doyle AE, Grevet EH, Grimm O, Halmoy A, Hoogman M, Hutz M, Jacob CP, Kittel-Schneider S, Knappskog PM, Lundervold AJ, Rivero O, Rovaris DL, Salatino-Oliveira A, da Silva BS, Svirin E, Sprooten E, Strekalova T, Adhd Working Group of the Psychiatric Genomics Consortium, team andMe Research, Arias-Vasquez A, Sonuga-Barke EJS, Asherson P, Bau CHD, Buitelaar JK, Cormand B, Faraone SV, Haavik J, Johansson SE, Kuntsi J, Larsson H, Lesch KP, Reif A, Rohde LA, Casas M, Borglum AD, Franke B, Ramos-Quiroga JA, Soler Artigas M, Ribases M (2020) Shared genetic background between children and adults with attention deficit/hyperactivity disorder. Neuropsychopharmacology 45:1617–26 10.1038/s41386-020-0664-5 PMC7419307 32279069

[CR48] Saviouk V, Hottenga JJ, Slagboom EP, Distel MA, de Geus EJ, Willemsen G, Boomsma DI (2011) ADHD in Dutch adults: heritability and linkage study. Am J Med Genet B Neuropsychiatr Genet 156B:352–62 10.1002/ajmg.b.31170 21294247

[CR49] Sawcer S, Jones HB, Judge D, Visser F, Compston A, Goodfellow PN, Clayton D (1997) Empirical genomewide significance levels established by whole genome simulations. Genet Epidemiol 14(3):223–9 9181352 10.1002/(SICI)1098-2272(1997)14:3<223::AID-GEPI1>3.0.CO;2-6

[CR50] Schanze I, Bunt J, Lim JWC, Schanze D, Dean RJ, Alders M, Blanchet P, Attie-Bitach T, Berland S, Boogert S, Boppudi S, Bridges CJ, Cho MT, Dobyns WB, Donnai D, Douglas J, Earl DL, Edwards TJ, Faivre L, Fregeau B, Genevieve D, Gerard M, Gatinois V, Holder-Espinasse M, Huth SF, Izumi K, Kerr B, Lacaze E, Lakeman P, Mahida S, Mirzaa GM, Morgan SM, Nowak C, Peeters H, Petit F, Pilz DT, Puechberty J, Reinstein E, Riviere JB, Santani AB, Schneider A, Sherr EH, Smith-Hicks C, Wieland I, Zackai E, Zhao X, Gronostajski RM, Zenker M, Richards LJ (2018) NFIB haploinsufficiency is associated with intellectual disability and macrocephaly. Am J Hum Genet 103:752–68 30388402 10.1016/j.ajhg.2018.10.006 PMC6218805

[CR51] Smalley SL, Kustanovich V, Minassian SL, Stone JL, Ogdie MN, McGough JJ, McCracken JT, MacPhie IL, Francks C, Fisher SE, Cantor RM, Monaco AP, Nelson SF (2002) Genetic linkage of attention-deficit/hyperactivity disorder on chromosome 16p13, in a region implicated in autism. Am J Hum Genet 71:959–63 12187510 10.1086/342732 PMC378550

[CR52] Sonuga-Barke EJS, Castellanos FX (2007) Spontaneous attentional fluctuations in impaired states and pathological conditions: a neurobiological hypothesis. Neurosci Biobehav Rev 31:977–86 17445893 10.1016/j.neubiorev.2007.02.005

[CR53] Spielman RS, McGinnis RE, Ewens WJ (1993) Transmission test for linkage disequilibrium: the insulin gene region and insulin-dependent diabetes mellitus (IDDM). Am J Hum Genet 52:506–16 8447318 PMC1682161

[CR54] Stahl K, Gola D, Konig IR (2021) Assessment of imputation quality: comparison of phasing and imputation algorithms in real data. Front Genet 12:724037 34630519 10.3389/fgene.2021.724037 PMC8493217

[CR55] Sudre G, Szekely E, Sharp W, Kasparek S, Shaw P (2017) Multimodal mapping of the brain’s functional connectivity and the adult outcome of attention deficit hyperactivity disorder. Proc Natl Acad Sci U S A 114:11787–92 29078281 10.1073/pnas.1705229114 PMC5676882

[CR56] Sudre, Gustavo, Luke Norman, Marine Bouyssi-Kobar, Jolie Price, Gauri Shastri, and Philip Shaw (2022) A mega-analytic study of white matter microstructural differences across five cohorts of youth with attention deficit hyperactivity disorder. Biol Psychiatry. 10.1016/j.biopsych.2022.09.021 PMC10039962 36609028

[CR57] Sudre G, Aman M, Philip S 2018) Growing out of attention deficit hyperactivity disorder: insights from the remitted’brain. Neurosci Behav Rev. 10.1016/j.neubiorev.2018.08.010 PMC9616204 30194962

[CR58] Sudre G, Frederick J, Sharp W, Ishii-Takahashi A, Mangalmurti A, Choudhury S, Shaw P (2020) Mapping associations between polygenic risks for childhood neuropsychiatric disorders, symptoms of attention deficit hyperactivity disorder, cognition, and the brain. Mol Psychiatry 25:2482–92 10.1038/s41380-019-0350-3 PMC6667324 30700802

[CR59] Sudre G, Saadia C, Eszter S, Teighlor B, Elanda G, Wendy S, Philip S (2017) Estimating the heritability of structural and functional brain connectivity in families affected by attention-deficit/hyperactivity disorder. JAMA Psychiatry 74:76–84 10.1001/jamapsychiatry.2016.3072 PMC7418037 27851842

[CR60] Sudre, Gustavo, Marine Bouyssi-Kobar, Luke Norman, Wendy Sharp, Saadia Choudhury, Philip Shaw (2021) Estimating the Heritability of Developmental Change in Neural Connectivity, and Its Association With Changing Symptoms of Attention-Deficit/Hyperactivity Disorder. Biological Psychiatry 89: 443−50 10.1016/j.biopsych.2020.06.007 PMC7736233 32800380

[CR61] Sutcubasi, Bernis, Baris Metin, Mustafa Kerem Kurban, Zeynep Elcin Metin, Birsu Beser, and Edmund Sonuga-Barke (2020) Resting-state network dysconnectivity in ADHD: a system-neuroscience-based meta-analysis. World J Biol Psychiatry 21:662−72 10.1080/15622975.2020.1775889 32468880

[CR62] Walton E, Pingault JB, Cecil CA, Gaunt TR, Relton CL, Mill J, Barker ED (2017) Epigenetic profiling of ADHD symptoms trajectories: a prospective, methylome-wide study. Mol Psychiatry. 22:250−56 10.1038/mp.2016.85 PMC5014094 27217153

[CR63] Wang K, Li M, Hakonarson H (2010) ANNOVAR: functional annotation of genetic variants from high-throughput sequencing data. Nucleic Acids Res 38:e164 10.1093/nar/gkq603 PMC2938201 20601685

[CR64] Wang Y, Gao W, Shi X, Ding J, Liu W, He H, Wang K, Shao F (2017) Chemotherapy drugs induce pyroptosis through caspase-3 cleavage of a gasdermin. Nature 547:99–103 10.1038/nature22393 28459430

[CR65] Watanabe K, Taskesen E, van Bochoven A, Posthuma D (2017) Functional mapping and annotation of genetic associations with FUMA. Nat Commun 8:1826 10.1038/s41467-017-01261-5 PMC5705698 29184056

[CR66] Wechsler D (2011) Wechsler Abbreviated Scale of Intelligence–Second Edition (WASI-II) San Antonio. *TX: Pearson.[Google Scholar]*.

[CR67] Whittemore AS, Halpern J (1994) A class of tests for linkage using affected pedigree members. Biometrics 50:118–27 8086596

[CR68] Wigginton JE, Abecasis GR (2005) PEDSTATS: descriptive statistics, graphics and quality assessment for gene mapping data. Bioinformatics 21:3445–7 15947021 10.1093/bioinformatics/bti529

[CR69] Wolraich ML, Lambert W, Doffing MA, Bickman L, Simmons T, Worley K (2003) Psychometric properties of the Vanderbilt ADHD diagnostic parent rating scale in a referred population. J Pediatr Psychol 28:559–67 14602846 10.1093/jpepsy/jsg046

[CR70] Yu D, Sul JH, Tsetsos F, Nawaz MS, Huang AY, Zelaya I, Illmann C, Osiecki L, Darrow SM, Hirschtritt ME, Greenberg E, Muller-Vahl KR, Stuhrmann M, Dion Y, Rouleau G, Aschauer H, Stamenkovic M, Schlogelhofer M, Sandor P, Barr CL, Grados M, Singer HS, Nothen MM, Hebebrand J, Hinney A, King RA, Fernandez TV, Barta C, Tarnok Z, Nagy P, Depienne C, Worbe Y, Hartmann A, Budman CL, Rizzo R, Lyon GJ, McMahon WM, Batterson JR, Cath DC, Malaty IA, Okun MS, Berlin C, Woods DW, Lee PC, Jankovic J, Robertson MM, Gilbert DL, Brown LW, Coffey BJ, Dietrich A, Hoekstra PJ, Kuperman S, Zinner SH, Luethvigsson P, Saemundsen E, Thorarensen O, Atzmon G, Barzilai N, Wagner M, Moessner R, Ophoff R, Pato CN, Pato MT, Knowles JA, Roffman JL, Smoller JW, Buckner RL, Willsey AJ, Tischfield JA, Heiman GA, Stefansson H, Stefansson K, Posthuma D, Cox NJ, Pauls DL, Freimer NB, Neale BM, Davis LK, Paschou P, Coppola G, Mathews CA, Scharf JM, (2019) the Gilles de la Tourette Gwas Replication Initiative, the Tourette International Collaborative Genetics Study, Tourette Association of America International Consortium for Genetics, Group the Psychiatric Genomics Consortium Tourette Syndrome Working Interrogating the genetic determinants of Tourette’s syndrome and other tic disorders through genome-wide association studies. Am J Psychiatry 176:217–27 10.1176/appi.ajp.2018.18070857 PMC6677250 30818990

[CR71] Zhou K, Asherson P, Sham P, Franke B, Anney RJ, Buitelaar J, Ebstein R, Gill M, Brookes K, Buschgens C, Campbell D, Chen W, Christiansen H, Fliers E, Gabriels I, Johansson L, Marco R, Mulas F, Muller U, Mulligan A, Neale BM, Rijsdijk F, Rommelse N, Uebel H, Psychogiou L, Xu X, Banaschewski T, Sonuga-Barke E, Eisenberg J, Manor I, Miranda A, Oades RD, Roeyers H, Rothenberger A, Sergeant J, Steinhausen HC, Taylor E, Thompson M, Faraone SV (2008) Linkage to chromosome 1p36 for attention-deficit/hyperactivity disorder traits in school and home settings. Biol Psychiatry 64:571–6 18439570 10.1016/j.biopsych.2008.02.024 PMC3589988

