# Supplementary material for: Combined family-based association and linkage analyses in families affected by attention-deficit hyperactivity disorder
Source: Hum Genet. 2026 Jun 22;145(1):52. doi: 10.1007/s00439-026-02840-7 (PMC13287277; doi:10.1007/s00439-026-02840-7)
Supplement: Supplementary file 1 — Supplementary Material 1 [file 439_2026_2840_MOESM1_ESM.docx]

**Supplemental Information**

**MRI Acquisition**

A baseline-weighted anatomical image [magnetization prepared rapid acquisition gradient recalled echo sequence (MP RAGE): 124 axial slices, 1.3-mm slice thickness, field of view = 22 cm, 224 × 224 acquisition matrix] was acquired on a 3-T General Electric MR 750 scanner. Multi-shell diffusion weighted data were acquired with a single-shot dual spin echo-planar imaging sequence with the following parameters: repetition time = 18.5 seconds, echo time = 85 ms, slice thickness = 2.5 mm, field of view = 240 mm, 96 × 96 matrix, gap = 0 mm, and acceleration factor = 2. A custom set of diffusion directions and weightings were acquired for a total of 60 volumes: 6 volumes at b value = 0 s/mm2, 12 volumes with evenly distributed directions at b value = 300 s/mm2, and 42 volumes with evenly distributed directions at b value = 1100 s/mm2. Up to two runs of resting-state fMRI data were acquired using a gradient-echo-planar series acquisition procedure (repetition time = 2,500 ms; echo time = 27 ms; slice thickness =2.8mm, flip angle = 90°; 44 axial contiguous interleaved slices per volume; field of view = 22 cm; 64 × 64 acquisition matrix; single-voxel volume = 3.4 mm, 3.4 mm, 2.8 mm, volumes=120), during which subjects were instructed to lie in the scanner at rest and look at a fixation cross.

**Preprocessing of neuroimaging data**

The fully automated and validated Freesurfer (version 5.3) "recon-all" pipeline was applied to MP RAGE images. The technical details of this pipeline and its associated procedures have been described in detail elsewhere (<http://surfer.nmr.mgh.harvard.edu/>). Following recommendations from previous work^1^, we excluded scans with a mean Euler number less than -217. After successful processing, the following FreeSurfer labels were extracted for further analyses:

Fractional anisotropy of left arcuate fasciculus

Fractional anisotropy of left anterior spinothalamic tract

Fractional anisotropy of left corpus callosum

Fractional anisotropy of right inferior fronto-occipital fasciculus

Fractional anisotropy of inferior longitudinal fasicuclus

Fractional anisotropy of superior longitudinal fasicuclus

Fractional anisotropy of left uncinate fasciculus

Total white matter area

Average cortical thickness

Right area of lateral orbitofrontal area

Right area of lateral superior frontal area

Right area of lateral fusiform thickness

Right area of lateral precentral thickness

Left amygdala volume

Right caudate volume

Total intracranial volume

Connectivity between default mode and dorsal attention networks

Connectivity between default mode and frontoparietal control networks

Connectivity between default mode and salience-ventral attention networks

Connectivity between default mode and somatomotor networks

DWI images were processed using QSIprep (https://qsiprep.readthedocs.io/en/latest/, version 0.8.0). Preprocessing steps included Gibbs unringing, B1 Bias field correction, head motion and eddy current correction. DWI images were aligned to the T1 space, and then transformed to the MNI152NLin2009cAsym template. Tensors were fitted using FSL’s dtifit and warped to the IIT Human Brain Atlas version 5.0 using DTI-TK (<http://dti-tk.sourceforge.net/pmwiki/pmwiki.php>). Masks for IIT atlas tracts were calculated using a threshold at the 5% of the maximum value of each fiber bundle file and applied to the skeletonized fractional anisotropy mask for each subject. The maximum mean framewise displacement among DTI images was 0.9. Resting-state data were preprocessed using fMRIPrep version 20.2.3, a Nipype based tool. Each T1w (T1-weighted) volume was corrected for intensity non-uniformity using N4BiasFieldCorrection v2.1.0 and skull-stripped using antsBrainExtraction.sh v2.1.0 (using the OASIS template). Brain tissue segmentation of cerebrospinal fluid, white-matter and gray-matter was performed on the brain-extracted T1w using fast (FSL 5.0.9, RRID:SCR_002823). Freesurfer’s recon-all was used to reconstruct brain surfaces (FreeSurfer 6.0.1, RRID:SCR_001847)^2^. The previously estimated brain mask was refined with a custom variation of the method to reconcile ANTs-derived and FreeSurfer-derived segmentations of the cortical gray-matter of Mindboggle (RRID:SCR_002438)^3^. Volume-based spatial normalization to MNI152NLin2009cAsym space was performed using brain-extracted versions of both T1w reference and the T1w template via nonlinear registration with antsRegistration (ANTs 2.3.3). Functional data were motion corrected using mcflirt (FSL v5.0.9)^4^. The remaining preprocessing steps used the xcpengine toolbox version 2.2.3 (https://xcpengine.readthedocs.io), and the 36-parameter + despiking functional design for deconvolution^5-9^. Despiking refers to the removal of and interpolation over intensity outliers in each voxel’s time series using AFNI’s 3DDESPIKE utility^10^. The 36-parameters regressed from the timeseries included the 6 motion estimates, global signal, and white matter and cerebrospinal fluid-derived time series), and then their derivatives, quadratic terms, and squares of derivatives^11,12^. Temporal filtering was performed using a bandpass filter of 0.01–0.08 Hz (first-order Butterworth filter^13^) and images were smoothed in FSL using a Gaussian-weighted kernel with 6 mm FWHM^14^.  Runs with mean-RMS ≤ 0.3 were concatenated using fslmerge to create a single time-series per subject and runs/subjects were excluded if they failed to complete the preprocessing pipeline or had mean-RMS >0.3. Regions of interest were taken from the Schaefer-400 atlas which has been mapped to the Yeo seven network parcellation^15,16^. Time courses from all voxels within a 5-mm spherical radius around each of the regions of interest were averaged and then correlated with each other. This created a single connectivity matrix per subject, which were subsequently Fisher-z transformed. Between network connectivity was calculated for each pairwise combination of networks by averaging Fisher-z scores of inter-network connections involving the relevant networks (e.g., all by averaging all cells for connections between a region of the default mode network default mode network and a region of the dorsal attention network).

References

1. Norman, L.J., Sudre, G., Bouyssi-Kobar, M., et al. (2021). A Longitudinal Study of Resting-State Connectivity and Response to Psychostimulant Treatment in ADHD. American Journal of Psychiatry Aug 1;178(8):744-751.
2. Dale, A.M., Fischl, B., Sereno, M.I. (1999). Cortical surface-based analysis. I. Segmentation and surface reconstruction. Neuroimage 9, 179-194.
3. Klein, A., Ghosh, S.S., Bao, F.S., Giard, J., Häme, Y., Stavsky, E., Lee, N., Rossa, B., Reuter, M., Chaibub Neto, E., et al. (2017). Mindboggling morphometry of human brains. PLoS Comput Biol. Feb 23;13(2):e1005350.
4. Jenkinson, M., Bannister, P., Brady, M., et al. (2002). Improved optimization for the robust and accurate linear registration and motion correction of brain images. Neuroimage 17:825–841.
5. Ciric, R., Wolf, D.H., Power, J.D., et al. (2017). Benchmarking of participant-level confound regression strategies for the control of motion artifact in studies of functional connectivity. Neuroimage; 154:174–187 25.
6. Ciric, R., Rosen, A.F.G., Erus, G., et al.(2018) Mitigating head motion artifact in functional connectivity MRI. Nat Protoc; 13:2801–2826 26.
7. Gur, R.E., Moore, T.M., Rosen, A.F.G., et al.(2019). Burden of Environmental Adversity Associated With Psychopathology, Maturation, and Brain Behavior Parameters in Youths. JAMA Psychiatry; 76:966–975 27.
8. Cui, Z., Li, H., Xia, C.H., et al. (2020). Individual variation in functional topography of association networks in youth. Neuron; 28.
9. Gu, S., Xia, C.H., Ciric, R., et al.(2020). Unifying the Notions of Modularity and Core–Periphery Structure in Functional Brain Networks during Youth. Cerebral Cortex; 30:1087– 1102.
10. Cox, R.W. (1996). AFNI: software for analysis and visualization of functional magnetic resonance neuroimages. Computers and Biomedical research; 29:162–173
11. Ciric, R., Wolf, D.H., Power, J.D., et al.(2017). Benchmarking of participant-level confound regression strategies for the control of motion artifact in studies of functional connectivity. Neuroimage; 154:174–187 25.
12. Ciric, R., Rosen, A.F.G., Erus, G., et al.(2018). Mitigating head motion artifact in functional connectivity MRI. Nat Protoc; 13:2801–2826
13. Hallquist, M.N., Hwang, K., Luna, B. (2013). The nuisance of nuisance regression: spectral misspecification in a common approach to resting-state fMRI preprocessing reintroduces noise and obscures functional connectivity. Neuroimage; 82:208–225
14. Jenkinson, M., Beckmann, C.F., Behrens, T.E., et al.(2012). Fsl. Neuroimage; 62:782–790
15. Yeo, B.T., Krienen, F M., Sepulcre, J., Sabuncu, M.R., Lashkari, D.,
    Hollinshead, M., et al. (2011). The organization of the human cerebral cortex
    estimated by intrinsic functional connectivity. J. Neurophysiol. 106, 1125–1165.
    doi: 10.1152/jn.00338.
16. Schaefer, A., Kong, R., Gordon, E.M., Laumann, T.O., Zuo, X.-N., Holmes,
    A.J., et al. (2018). Local-global parcellation of the human cerebral cortex from
    intrinsic function. Sep 1;28(9):3095-3114.

**Supplemental Figures and Legends**

**Figure S1.** Manhattan and quantile-quantile plots for ADHD analysis of (a) NHGRI Family Cohort; (b) NCR Family Cohort. The horizontal blue line corresponds to *P* < 1 x 10^-5^, while the horizontal red line corresponds the genome-wide significant threshold of *P* < 5 x 10^-8^.

**
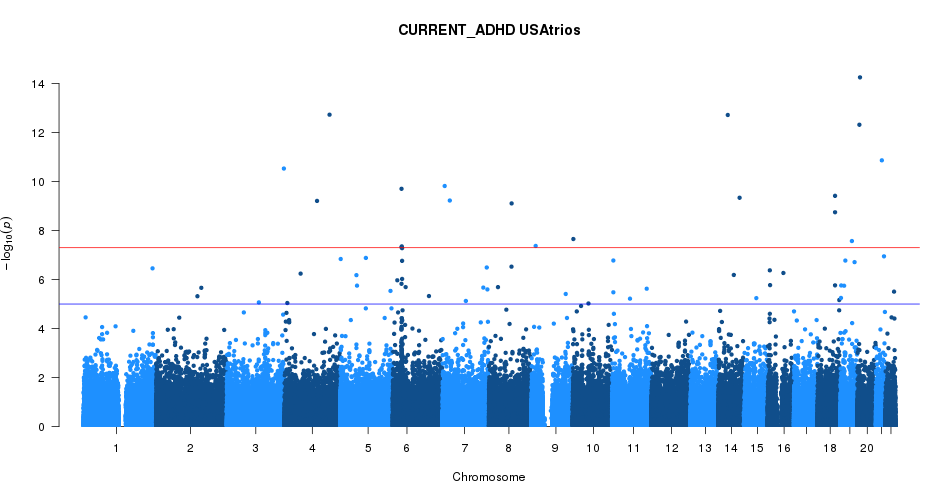
**

(a)


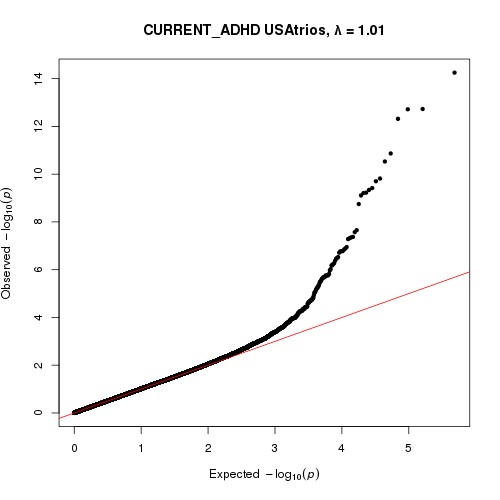


λ=1.01

**
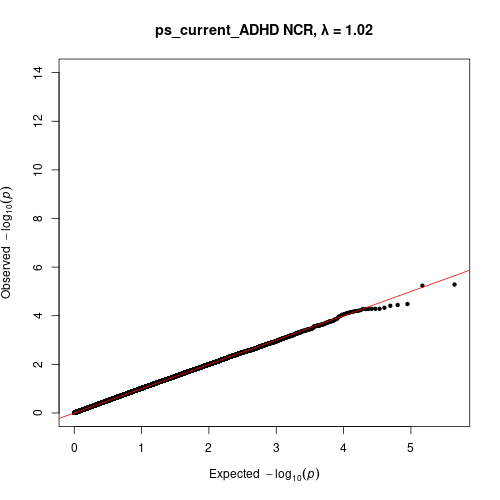

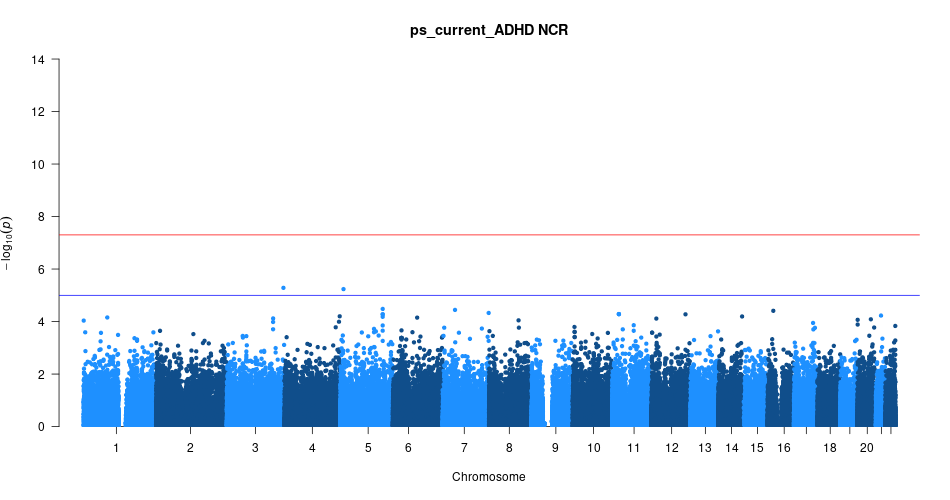
**

λ=1.02

(b)

**Figure S2: Regional association plots for meta-analysis association at genome-wide significance on (a) chromosome 3, (b) chromosome 6 and (c) chromosome 7**. *P* values (−log_10_) of the meta-analysis are plotted against the genomic positions of each SNP, with genes in the region shown below. The linkage disequilibrium values (*r*^2^) between the lead SNP and other SNPs are indicated in different colors


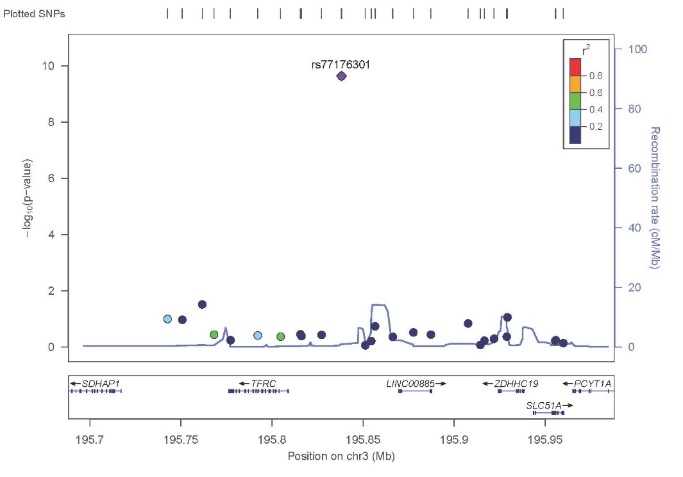


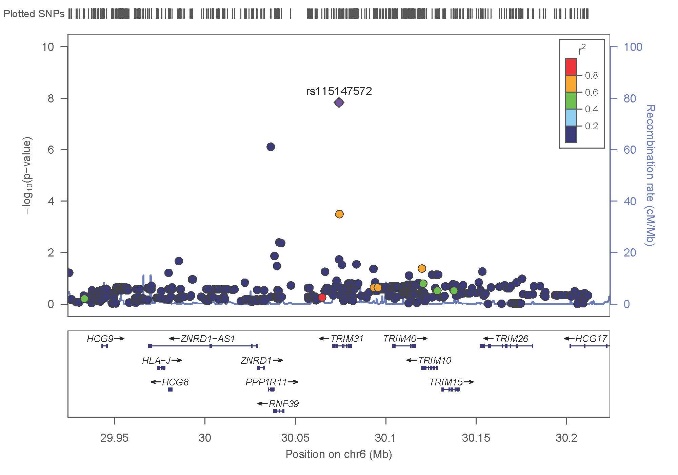


**a.**

**b.**


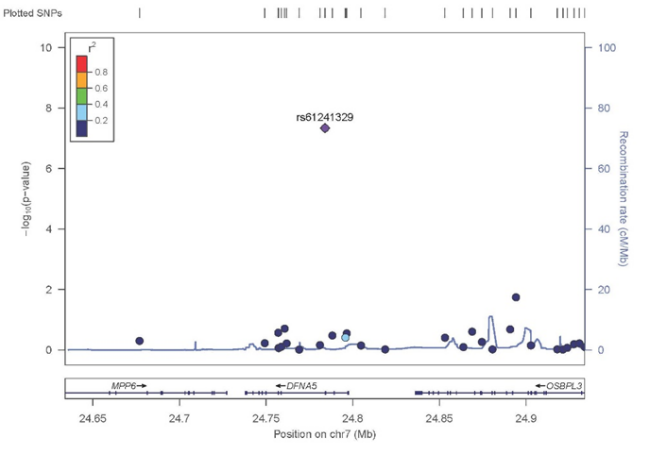


**c.**

**Figure S3.** Genome-wide plot of the linkage p-values (−log_10_) of the NGRI Family Cohort. The horizontal blue line corresponds to the suggestive linkage threshold (LOD > 2.0, *P* < 0.001), while the horizontal red line corresponds the significant linkage score threshold (LOD > 3.0, *P* < 0.0001).


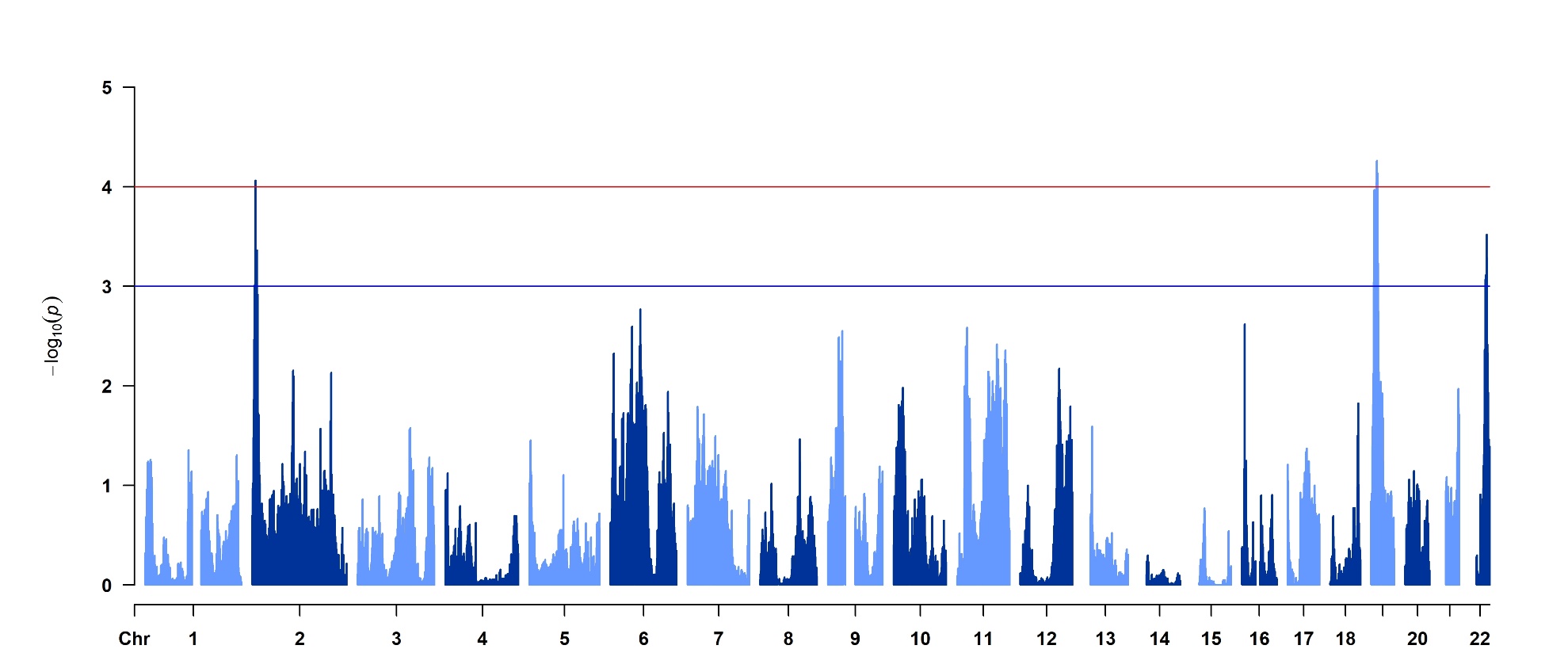


**Figure S4.** Genome-wide plot of the linkage p-values (−log_10_) of the NCR Family Cohort. The horizontal blue line corresponds to the suggestive linkage threshold (LOD > 2.0, *P* < 0.001), while the horizontal red line corresponds the significant linkage score threshold (LOD > 3.0, *P* < 0.0001).


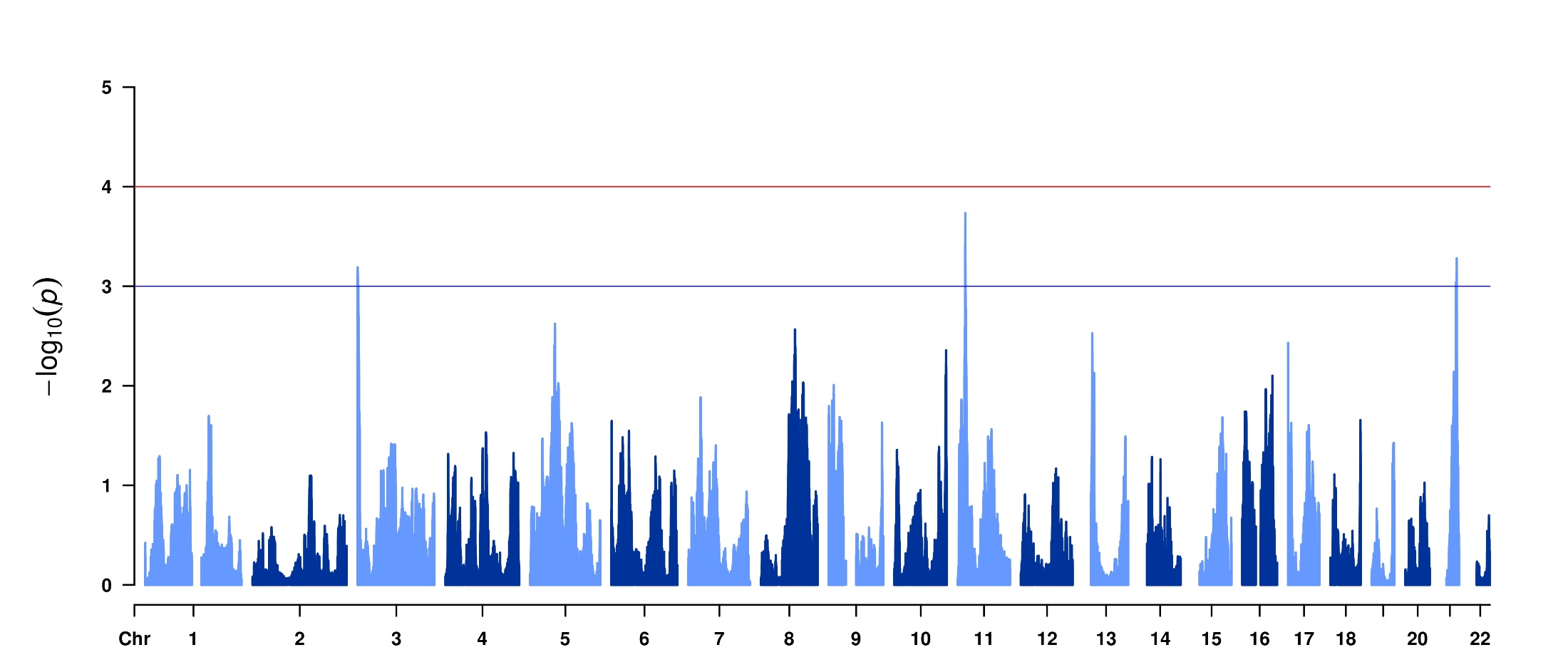


**Figure S5.** Manhattan plot of gene-based association test for ADHD analysis of NHGRI Family Cohort.


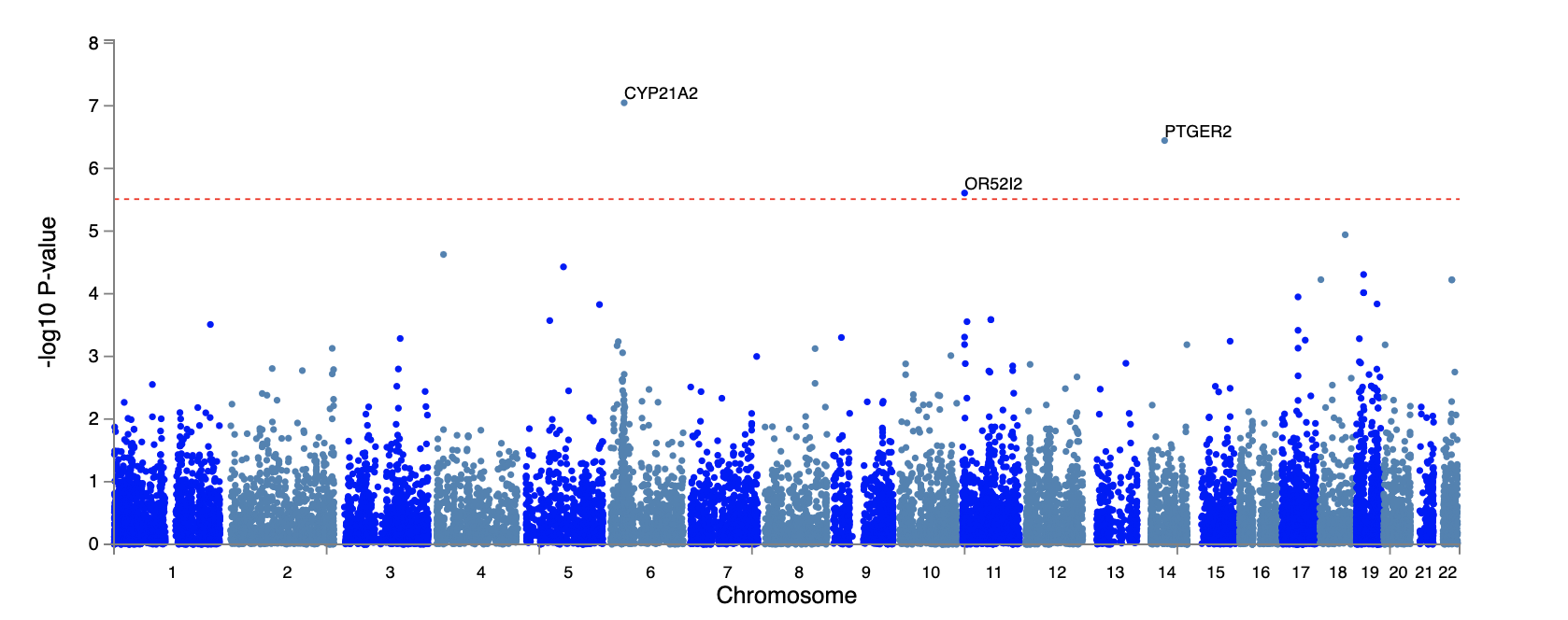


**Figure S6.** Manhattan plot of gene-based association test for ADHD analysis of meta-analysis of NHGRI Family Cohort and NCR Family Cohort.


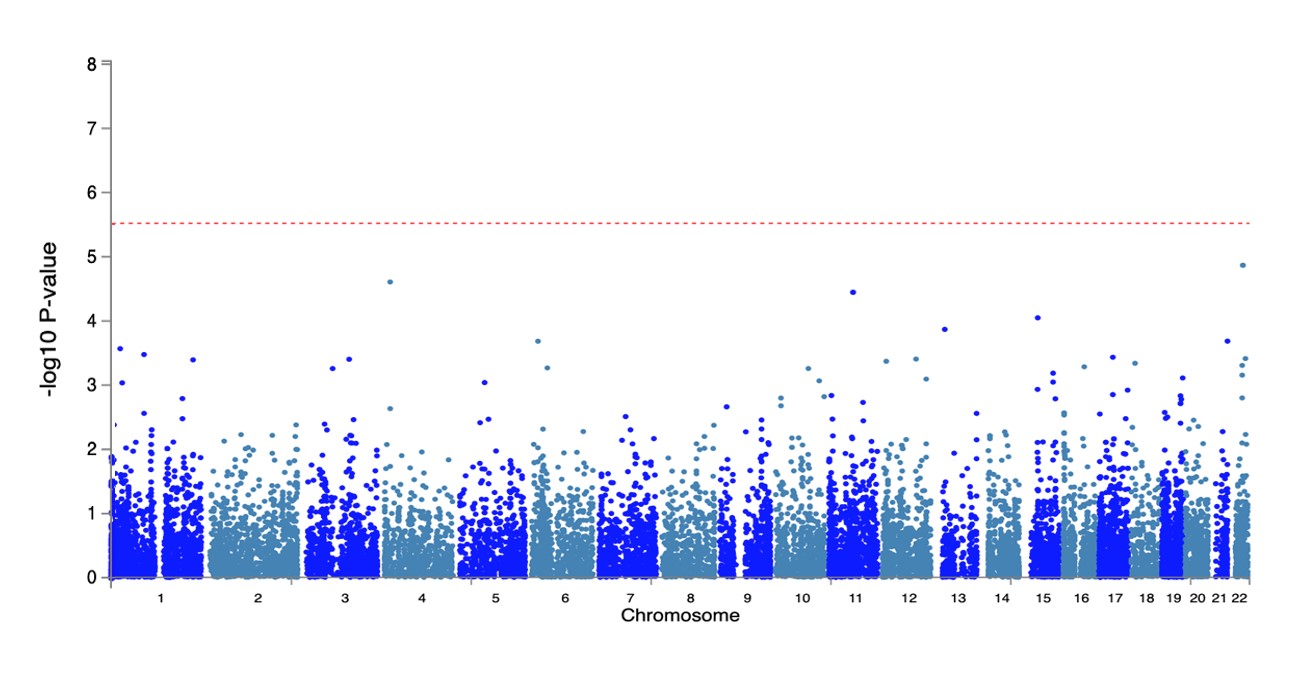


| **Supplemental Tables**  **Table S1**. List of comorbidities that excluded proband from being enrolled in the NHGRI Family Cohort |
| --- |
| Tourette's disorder |
| Obsessive-compulsive disorder |
| Pervasive developmental disorders |
| Psychotic disorders |
| Mood disorders with psychotic features |
| Post-traumatic stress disorder |
| Previous diagnosis of lead toxicity |
| Neurological conditions |
| Known genetic syndromes |
| Mental retardation |
| Hydrocephaly |
| Known prenatal drug exposure |
| Cardiac surgery |
| Prematurity (birth weight < 2,500 grams) |
| Major depression if proband and sibling had lifetime history |

**Table S2**. Curated gene list used for GSEA analysis

| Gene set | Reference | Genes |
| --- | --- | --- |
| ADHD_CNV | Harich et al., 2020 | COPA,CPSF2,CSMD1,DMRTB1,MAPK1,MSH3,MYC,NCSTN,NDUFA5,NDUFB1,PARK2,PDCD6IP,PEA15,PLOD3,POLR1A,POLR2B,POLR3C,PPM1F,  RBFOX1,SEPT5,TBL1XR1,TTC27,TUBA3C,TUBGCP2,WASL,WWOX |
| ADHD_GWAS_2022 GWAS hits | Demontis et al., 2022 | PTPRF,KDM4A,SNRK,ANO10,ABHD5,TRAIP,CAMKV,MST1R,CTD2330K9.3,MON1A,IQCF3,IQCF2,IQCF5,IQCF1,FOXP1,VGLL3,LSM6,RP11‑6L6.2,  SLC10A7,COL19A1,FOXP2,C8orf82,ARHGAP39,SORCS3,METTL15,DUSP6,POC1B,CDH8,TMEM200C,DCC,XRN2,NKX2-4 |
| ADHD_GWAS_2022 76 risk genes | Demontis at al., 2022 | PPIH,C1orf210,ELOVL1,MED8,SZT2,HYI,PTPRF,ST3GAL3,ARTN,B4GALT2,KAT2B,FAM198A,SNRK,ANO10,ABHD5,ZNF852,ZNF660,ZNF197,GPX1,  RHOA,TCTA,AMT,MST1,RNF123,AMIGO3,GMPPB,FAM212A,TRAIP,CAMKV,MST1R,CTD‑2330K9.3,MON1A,RBM6,GNAT1,GNAI2,HYAL3,MAPKAPK3,IQCF3,FOXP1,VGLL3,ZNF827,LSM6,RP11‑6L6.2,SH3RF2,COL19A1,FOXP2,FAM83H,FAM203A,MROH1,VPS28,CYHR1,KIFC2,PPP1R16A,MFSD3,RECQL4,LRRC14,LRRC24,C8orf82,ARHGAP39,ZNF251,ZNF34,RPL8,ZNF517,ZNF7,COMMD5,ZNF16,SORCS3,POC1B,VRK1,CDH8,TMEM200C,L3MBTL4,ARHGAP28,DCC,XRN2,NKX2-4 |
| ADHD_TWAS_Gustavo | Sudre et al., 2022 | HAMP,CCL2,CRISPLD1,HILPDA,MYO1G,JPH2,ZNF256,PDPN,ADAMTS9,ANGPTL4,KIAA0040,CCDC13 |
| ASD_GWAS_2019 | Grove et al., 2019 | KIZ,C8orf74,LOC102723661,MACROD2,KMT2E,MMS22L,NUD12,MROH5,CADPS,NEGR1,MARK3,SLC30A9,XRN2,NKX2‑2,NKX24,SOX7,PINX1,PTBP2,  SRPK2,POU3F2,CKB,TRMT61A,BAG5,APOPT1,KLC1,XRCC3,BEND4,TMEM33,DCAF4L1 |
| SCZ_TWAS_2018 | Gusev et al., 2018 | HIST1H4L,TRIM27,HIST1H4J,HIST1H3C,RP5‑874C20.3,ZSCAN12P1,HIST1H1A,RP11‑272L13.4,PCGF6,HIST1H2AD,ZSCAN23,SETD8,CNNM2,  RP1‑265C24.5,C2orf47,BTN3A2,ZNF192P2,AL022393.7,RP1‑313I6.12,MPHOSPH9,GATAD2A,RP11_73M18.2,ZKSCAN3,MAU2,AC110781.3,SF3B1,ZNF184,FXR1,U91328.22,ZKSCAN8,INO80E,MDK,TSNARE1,TMEM219,HCG11,FAM109B,NAGA,SNAP91,ZNF165,HIST1H4H,CYP17A1‑AS1,VPS45,VPS29,  LINC00634,CTA‑14H9.5,PAK6,RPS17L,ETF1,RP11‑231C14.4,NPM1P19,DGKI,BAG5,RP11‑282O18.3,EFTUD1P1,PPP1R13B,PSMA4,RPS17,NDFIP2,WDR46,ZNF192P1,PCCB,DAXX,THOC7,GIGYF1,RP11‑350N15.5,SETD6,ARL6IP4,PSMB8,RP11‑231C14.3,ZC3H7B,IP6K3,BTN2A2,CYP2D6,DDHD2,HLA‑DOA,  RP11‑182J1.13,AS3MT,WDR82,APOPT1,CTA‑250D10.23,FTSJ2,GLYCTK,OGFOD2,ALMS1P,SNORD3B2,RP11‑182J1.14,RP11‑182J1.10,HYKK,NSG1,  TPRKB,KMT2E,PLCL1,SNX19,PACSIN3,HIST1H3A,CACNA1D,C2orf16,DCLK3,MCHR1,RP11‑182J1.16,EMB,ZNF804A,CTB‑152G17.6,RP11‑197N18.2,  SGOL2,DNAJA3,NAT8,ZSCAN16,FURIN,JKAMP,TOM1L2,RERE,RP11‑73M18.8,DARS2,RP1‑95L4.4,RP11‑380L11.3,C9orf131,ZMAT2,CSPG4P11,  SDCCAG8,AC011330.5,ASAP1,HNRNPA1P46,ASPHD1,ZNF391,JRK,MPPED2,TYW5,C17orf75,GID4,PCDHA8,CYP2D7P1,AKT3,LRRC37A2,GNL3,  DNAJC11,RP11‑894P9.1,RP11‑247A12.7,SLC9C2,RP11‑245J9.5,XRCC3,ELAC2,CLEC18B,AL049840.1,RP11‑53O19.3,LRRC37A,HLA‑DPA1,NDUFA6‑AS1,  ARL17A,RP13‑262C2.3,RP11‑451F14.1,AC145123.2,FAM114A2,CENPM,COG8,HAPLN4,GNL3LP1,TSNAXIP1,MARS2,VPS4A,TMEM214,CMAHP,  SLC25A12,HMGN4,LINC00933,CTD‑2270N23.1,TAPBP,DCC,RP11‑239L20.6,ZNF513,C16orf52,CDK2AP1,RP11‑499P20.2,MAPK3,BAK1,STAB1,ATG13,  RP5‑1112D6.7,TMED4,CTSS,RP117O11.3,XPNPEP3,PCDHA7,PPP2R5B,NIP7,RP11‑247A12.2,PDF,WBP1L,RANGAP1,ZSCAN2,IGSF9B,SEPT10,  PBXIP1 |
| SCZ_GWAS_2018 | Schizophrenia Working Group of the PGC, 2018 | ABCB1,ABCB4,ABCB9,ABCD2,AC005477.1,AC005609.1,AC027228.1,AC073043.2,ACD,ACO2,ACP2,ACTR1A,ACTR5,ADAMTS7P1,ADAMTSL3,  ADAMTSL4,ADAMTSL4‑AS1,ADM5,AGPHD1,AIG1,AKAP6,AKT3,AL049840.1,AL050132,ALAS1,ALDOA,ALMS1,ALMS1‑IT1,ALMS1P,ALPK3,  AMBRA1,ANAPC7,ANKRD13A,ANKRD44,ANKRD44‑IT1,ANKRD45,ANKRD63,ANP32E,ANXA9,AP3B2,AP5B1,APH1A,APOPT1,ARFGAP2,ARHGAP1,  ARHGAP40,ARL14EP,ARL3,ARL5B,ARL6IP4,ARNT,ARPC3,ARTN,AS3MT,ASCL1,ASH2L,ASPG,ASPHD1,ATF4,ATG13,ATP13A1,ATP2A2,ATP6V0B,  ATPAF2,ATXN7,B3GAT1,B4GALT2,B9D1,BAG4,BAG5,BANK1,BAP1,BC037927,BCL11B,BCL2L12,BNIP3L,BNIPL,BOLL,BRD8,BRINP2,BTBD18,BTG1,  C10orf32,C10orf32‑ASMT,C11orf31,C11orf49,C11orf87,C12orf42,C12orf65,C12orf76,C12orf79,C14orf2,C16orf86,C16orf92,C1orf132,C1orf51,C1orf54,C1orf56,C22orf46,C2orf47,C2orf69,C2orf78,C2orf82,C3orf49,C4orf27,CA14,CA8,CACNA1C,CACNA1C‑AS4,CACNA1C_IT3,CACNA1D,CACNA1I,CACNB2,  CACYBP,CALB2,CALHM1,CALHM2,CALHM3,CCDC134,CCDC175,CCDC24,CCDC39,CCDC62,CD14,CD46,CDC20,CDC23,CDC25C,CDC42SE1,  CDK2AP1,CENPL,CENPM,CENPT,CEP170,CERS2,CHADL,CHRM3,CHRM4,CHRNA2,CHRNA3,CHRNA5,CHRNB4,CHST4,CIART,CILP2,CKAP5,CKB,  CLCN3,CLDN23,CLP1,CLU,CMTR2,CNKSR2,CNNM2,CNOT1,CNTN4,COQ10B,CPEB1,CPNE8,CPT1C,CR1L,CREB3L1,CROT,CSDC2,CSMD1,CTB‑12O2.1,CTC‑436P18.1,CTNNA1,CTNND1,CTRL,CTSK,CTSS,CUL3,CYP17A1,CYP26B1,CYP2D6,CYP2D7P,DAAM1,DARS2,DCP1A,DDB2,DDHD2,DDX28,  DEPDC1B,DESI1,DFNA5,DGKI,DGKZ,DMTF1,DNAH1,DNAJB7,DNAJC19,DND1,DNM1P41,DOC2A,DOPEY1,DPEP2,DPEP3,DPH2,DPP4,DPYD,  DPYD‑AS1,DPYD‑AS2,DRD2,DRG2,DUS2,DUS2L,DUSP11,ECM1,EDC4,EFHD1,EFTUD1,EFTUD1P1,EGR1,EGR4,EHBP1L1,EIF5,ELOVL1,ELOVL7,EMB,EMX1,ENKD1,ENSA,EP300,EP300‑AS1,EPC2,EPHX2,EPN2,EPN2‑AS1,EPN2‑IT1,ERCC4,ERCC8,ERI1,ESAM,ESRP2,ETF1,F2,FAM109B,FAM154B,  FAM216A,FAM53C,FAM57B,FAM5B,FAM63A,FAM86B3P,FANCL,FES,FGFR1,FHIT,FLJ31662,FOXG1,FOXP1,FSD2,FSHB,FTCDNL1,FTSJ2,FURIN,FUT9,FXR1,GABBR2,GABPB2,GALNT10,GAS5,GAS5‑AS1,GATAD2A,GDPD3,GFOD2,GFRA3,GID4,GIGYF2,GIT2,GLT8D1,GLTP,GLYCTK,GMIP,GNL3,  GOLGA2P7,GOLGA6L10,GOLGA6L17P,GOLGA6L4,GOLGA6L5P,GOLGA6L9,GOLPH3L,GPM6A,GPN3,GPR135,GPR182,GPR52,GRAMD1B,GRAP,  GRAPL,GRIA1,GRIN2A,GRM3,HAPLN4,HARBI1,HARS,HARS2,HCN1,HIP1R,HIRIP3,HOMER2,HORMAD1,HS3ST5,HSPA9,HSPD1,HSPE1,HSPE1‑MOB4,HVCN1,HYDIN,HYI,HYKK,IFT81,IGSF9B,IK,IL20RB,IMMP2L,INA,INHBC,INO80E,INPP4B,IPO13,IREB2,IRF3,ITIH1,ITIH3,ITIH4,JKAMP,KAT5,KCNB1,KCNG2,KCNJ13,KCNK7,KCNV1,KCTD13,KDM3B,KDM4A,KDM4A‑AS1,KIAA1324L,KIF20A,KIF21A,KLC1,KLHL20,KMT2E,KMT2E‑AS1,L3HYPDH,  L3MBTL2,LCAT,LETM2,LHFPL3,LHFPL3‑AS2,LINC00051,LINC00461,LINC00568,LINC00599,LINC00606,LINC00634,LINC00637,LINC00862,LINC00933,LINC01004,LINC01288,LOC100131303,LOC100505474,LOC100506472,LOC100506674,LOC100507091,LOC101243545,LOC101927134,LOC101927229,  LOC101927273,LOC101927295,LOC101927641,LOC101927839,LOC101928441,LOC101928782,LOC101928882,LOC101929406,LOC101929829,  LOC102546226,LOC102723362,LOC102724034,LOC103171574,LOC157273,LOC283177,LOC283692,LOC283693,LOC338963,LOC388436,LOC388906,  LOC440300,LOC440704,LOC642423,LOC727751,LOC730159,LOC731275,LOC79999,LOC80154,LPAR2,LRP1,LRP4,LRP4‑AS1,LRRC48,LRRIQ3,LRRN3,  LSM1,LUZP2,MAD1L1,MADD,MAN2A1,MAN2A2,MAP3K11,MAPK3,MAPK7,MARK3,MARS2,MAU2,MCHR1,MCL1,MDK,ME1,MED19,MED8,MEF2C,  MEF2C‑AS1,MEI1,MAP4,MFHAS1,MGAT3,MIEF1,MIR1180,MIR1228,MIR124‑1,MIR1281,MIR1284,MIR1307,MIR130A,MIR135A1,MIR137,MIR137HG,  MIR2682,MIR3160‑1,MIR3160‑2,MIR33A,MIR33B,MIR3714,MIR4257,MIR4301,MIR4304,MIR4472‑1,MIR4489,MIR4497,MIR4529,MIR4655,MIR4660,  MIR4677,MIR4688,MIR4690,MIR4766,MIR5088,MIR548AI,MIR548AJ2,MIR5582,MIR597,MIR6079,MIR6734,MIR6735,MIR6745,MIR6773,MIR6777,  MIR6836,MIR6842,MIR6843,MIR6854,MIR6878,MIR6889,MIR8064,MIR8072,MIR9‑2,MIRLET7G,MKL1,MLL5,MLLT11,MMP16,MOB4,MPHOSPH9,MPL,  MPP6,MPPED2,MRPS14,MRPS21,MRPS30,MSANTD2,MSL2,MSRA,MUSTN1,MYBPC3,MYO15A,MYO1A,NAB2,NAGA,NAT8,NAT8B,NCAN,NCK1,  NCK1‑AS1,NDFIP2,NDFIP2‑AS1,NDRG4,NDUFA13,NDUFA2,NDUFA4L2,NDUFA6,NDUFA6‑AS1,NDUFAF2,NEK1,NEK4,NEURL1,NFATC3,NGEF,  NHP2L1,NISCH,NLGN4X,NMB,NME5,NMUR2,none,NOSIP,NR1H3,NRGN,NRN1L,NSUN6,NT5C2,NT5DC2,NUDT1,NUTF2,NXPH4,OGFOD2,OPCML,  OSBPL3,OTOL1,OTUD7B,OVOL1,OVOL1‑AS1,PACSIN3,PAK6,PARD6A,PBRM1,PBX4,PCCB,PCDHA1,PCDHA10,PCDHA2,PCDHA3,PCDHA4,PCDHA5,  PCDHA6,PCDHA7,PCDHA8,PCDHA9,PCGEM1,PCGF6,PCNXL3,PDCD11,PDE4B,PGM3,PHF5A,PHF7,PITPNM2,PJA1,PLA2G15,PLCB2,PLCH2,PLCL1,  PLCL2,PLEKHO1,PMM1,PODXL,POLR3H,PPAPDC1B,PPM1M,PPP1CC,PPP1R13B,PPP1R16B,PPP1R3B,PPP2R2A,PPP2R3A,PPP4C,PPTC7,PRKCB,PRKCD,PRKD1,PRMT1,PRMT7,PRPF3,PRR12,PRRG2,PRSS35,PRUNE,PSD3,PSKH1,PSMA4,PSMB10,PSMD6,PSMD6‑AS2,PTGIS,PTK2B,PTN,PTPRF,PTPRK,PUS7,R3HDM2,RABGAP1L,RAD9B,RAI1,RALGAPA2,RANBP10,RANGAP1,RBFOX1,RBM26,RBM26‑AS1,RBX1,RC3H1,RCN3,RD3L,RDH16,REEP2,RELA,  RERE,RFT1,RFTN2,RGS6,RILPL2,RIMS1,RLTPR,RNASEH2C,RNF112,RPEL1,RPRD2,RPS17,RPS19BP1,RPTOR,RRAS,RTN1,RUNDC3B,RWDD2A,SATB2,SBNO1,SCAF1,SCAND2P,SCARNA15,SDCCAG8,SEC11A,SEMA3G,SEMA6C,SEMA6D,SEPT3,SERPINC1,SERPING1,SETD6,SETD8,SETDB1,SEZ6L2,  SF3B1,SFMBT1,SFXN2,SFXN5,SGK223,SGSM2,SHISA8,SHMT2,SIPA1,SLC12A4,SLC25A17,SLC32A1,SLC35G2,SLC38A7,SLC39A8,SLC45A1,SLC4A10,  SLC5A10,SLC6A11,SLC6A9,SLC7A6,SLC7A6OS,SLC9C2,SMCR5,SMDT1,SMG6,SMIM15,SMIM4,SMPD3,SNAP91,SNHG21,SNORA28,SNORA46,  SNORA76A,SNORD19,SNORD19B,SNORD44,SNORD47,SNORD63,SNORD67,SNORD69,SNORD74,SNORD75,SNORD76,SNORD77,SNORD78,SNORD79,  SNORD80,SNORD81,SNORD91A,SNORD91B,SNX19,SNX8,SOX2‑OT,SOX5,SPATA31D1,SPATA31D3,SPATA31D4,SPATA31D5P,SPATS2L,SPCS1,  SREBF1,SREBF2,SRPK2,SRR,ST13,ST3GAL3,STAB1,STAC3,STAG1,STAMBP,STAR,STAT6,SUFU,SUGP1,SZT2,TAB1,TAC3,TAF5,TAOK2,TARS2,  TBC1D2,TBC1D5,TBX6,TCF20,TCF4,TCHP,TCTN1,TDRD9,TEF,THAP11,THOC7,THOC7‑AS1,TKT,TLE1,TLE3,TLR9,TM6SF2,TMCO6,TMEM110,  TMEM110‑MUSTN1,TMEM161B,TMEM161B‑AS1,TMEM180,TMEM194A,TMEM219,TMEM243,TMTC1,TMX2,TMX2‑CTNND1,TNFRSF13C,TNKS,  TNNC1,TOB2,TOM1L2,TP53TG1,TPRKB,TRANK1,TRIM8,TRMT61A,TRPV4,TSNARE1,TSNAXIP1,TSR1,TSSK6,TWF2,TYW5,U80770,UBE2Q2L,  UBE2Q2P1,UBE2Q2P2,UBE3D,USMG5,VPS13C,VPS14C,VPS29,VPS37B,VPS45,VRK2,VSIG2,WBP1L,WBP2NL,WDR55,WDR73,WDR82,WHAMM,  WHSC1L1,xMHC,XPNPEP3,XRCC3,XRCC6,YJEFN3,YPEL3,YPEL4,ZBTB37,ZBTB39,ZC3H7B,ZDHHC5,ZEB2,ZFYVE21,ZMAT2,ZNF101,ZNF19,ZNF23,  ZNF281,ZNF408,ZNF440,ZNF441,ZNF491,ZNF536,ZNF592,ZNF804A,ZNF823,ZSCAN2,ZSWIM6 |
| BD_GWAS_2019 | Stahl at al., 2019 | PLEKHO1,LMAN2L,SCN2A,PCGEM1,TRANK1,ITIH1,CD47,FSTL5,ADCY2,SSBP2,RIMS1,POU3F2,RPS6KA2,THSD7A,SRPK2,MRPS33,ANK3,ADD3,FADS2,PACS1,PC,CACNA1C,STARD9,ZNF592,GRIN2A,HDAC5,ZCCHC2,NCAN,STK4,TFAP2B,DFNA5,SLC25A17,HLF,PHF15,ODZ4,VRK2,IMMP2L |

**Table S3.** Genome-wide significant (*P* < 5 x 10-8) results for association analysis of the NHGRI Family Cohort, ADHD, ADHD+ODD and ADHD only

| CHR | BP | SNP | A1 | A2 | MAF | *P*-values | | | Location | Gene |  |
| --- | --- | --- | --- | --- | --- | --- | --- | --- | --- | --- | --- |
|  |  |  |  |  |  | ADHD | ADHD+ODD | ADHD only |  |  |  |
| 3 | 195838160 | rs77176301 | C | T | 0.059 | 2.95E-11 | 2.58E-14 | 8.490E-07 | upstream | *LINC00885, TFRC* |  |
| 4 | 111707130 | rs62337211 | A | C | 0.15 | 6.17E-10 | 1.94E-09 | 7.820E-07 | intergenic | *PITX2, MIR297* |  |
| 4 | 154603296 | rs2405433 | T | C | 0.19 | 1.87E-13 | 1.28E-12 | 1.710E-08 | upstream; downstream | *TLR2, LOC100419170, RNF175, TMEM131L* |  |
| 6 | 30036333 | rs17187693 | C | G | 0.29 | 4.81E-08 |  | 2.640E-06 | intronic | *PPP1R11* |  |
| 6 | 30074086 | rs115147572 | A | C | 0.11 | 1.98E-10 | 9.46E-10 | 4.150E-07 | intronic | *TRIM31* |  |
| 6 | 31045554 | rs9378152 | G | C | 0.13 | 4.43E-08 | 3.37E-09 | 0.000198 | upstream; downstream | *PSORS1C1, C6orf15, CDSN, HCG22, MUC22* |  |
| 6 | 32019382 | rs115521560 | C | A | 0.05 |  | 3.24E-08 | 0.000019 | intronic | *TNXB* |  |
| 6 | 32096244 | rs3830076 | T | C | 0.06 |  | 3.43E-08 | 0.002304 | upstream; downstream | *ATF6B, FKBPL* |  |
| 7 | 7223618 | rs60600046 | G | A | 0.06 | 1.52E-10 | 2.07E-11 | 3.140E-06 | intronic | *C1GALT1* |  |
| 7 | 24783987 | rs61241329 | A | C | 0.07 | 5.95E-10 | 9.76E-11 | 1.250E-06 | intronic | *GSDME* |  |
| 8 | 77927157 | rs536249779 | T | G | 0.12 | 7.81E-10 | 7.51E-10 | 4.890E-06 | upstream | *MIR3149, PEX2* |  |
| 9 | 14246998 | rs10961448 | C | A | 0.08 | 4.25E-08 |  | 0.000231 | intronic | *NFIB* |  |
| 10 | 2742533 | rs7911061 | A | G | 0.16 | 2.22E-08 | 1.55E-08 | 9.300E-06 | intergenic | *LINC02645, LOC101927824* |  |
| 14 | 52778656 | rs5003100 | A | G | 0.08 | 1.92E-13 | 2.97E-13 | 9.220E-09 | upstream; downstream | *PTGER2, PTGDR* |  |
| 14 | 93263982 | 14:93263982 | C | G | 0.16 | 4.58E-10 | 4.60E-11 | 0.000125 | exonic | *GOLGA5* |  |
| 16 | 8356326 | rs17145695 | C | T | 0.06 |  | 4.66E-08 | 0.000703 | intergenic | *RBFOX1, TMEM114* |  |
| 18 | 59994265 | rs8087597 | G | A | 0.15 | 3.82E-10 | 1.36E-09 | 6.810E-09 | intronic | *TNFRSF11A* |  |
| 18 | 60024128 | rs58112300 | C | T | 0.06 | 1.79E-09 | 2.06E-09 | 3.700E-06 | intronic | *TNFRSF11A* |  |
| 19 | 39993470 | rs55741253 | T | A | 0.24 | 2.68E-08 |  | 5.150E-06 | exonic | *DLL3* |  |
| 20 | 6697162 | rs34169461 | T | A | 0.11 | 4.80E-13 | 5.63E-14 | 9.540E-07 | downstream | *LINC01713* |  |
| 20 | 8817556 | rs58568270 | G | A | 0.1 | 5.61E-15 | 4.95E-14 | 1.770E-09 | intronic | *PLCB1* |  |
| 21 | 35715023 | rs73902815 | C | T | 0.06 | 1.36E-11 | 8.02E-11 | 6.870E-09 | upstream | *KCNE2, SMIM11A, SMIM11B* |  |
| CHR, chromosome; BP, base pair position (GRCh37/hg19); A1, minor allele in NHGRI Family cohort; A2, major allele in NHGRI Family cohort; MAF, minor allele frequency | | | | | | | | | | |  |
|  |  |  |  |  |  |  |  |  |  |  |  |

**Table S4.** Previous linkage studies reporting LOD scores > 3.0

| Study | Ogdie et al. 2003 | Bakker et al. 2003 | Arcos-Burgos et al. 2004 | Hebebrand et al. 2006 | Asherson et al. 2008 | Romanos et al. 2008 | Saviouk et al. 2011 |
| --- | --- | --- | --- | --- | --- | --- | --- |
| Ethnicity | Caucasian and African American | Caucasian | Paisa decent | Caucasian | Caucasian | Caucasian | Caucasian |
| Population | USA | Dutch | Columbia | German | 8 European countries | German | Dutch |
| Sample size | 204 families | 164 sib pairs | 18 families | 102 families | 134 families | 8 families | 750 sib pairs |
| Analysis program | Mapmaker/sibs | Mapmaker/sibs | FASTLINK | Merlin | Merlin | Genehunter | Merlin |
| Region with LOD > 3 (LOD) | 16p13(3.73) | 7p13 (3.04) | 4q13.2 (4.44) | 5p15(3.35) | 16q23 (LOD 3.1) | 9q22(3.10) | 2p25.1(3.58) |
|  |  | 15q15.1(3.21) | 5q33.3 (8.22) |  |  | 16q24.1(3.57) | 18q21(4.58) |
|  |  |  | 11q22 (5.77) |  |  |  |  |
|  |  |  | 17p11 (3.73) |  |  |  |  |

| **Table S5.** PRS analysis results | | | | | | | |
| --- | --- | --- | --- | --- | --- | --- | --- |
| PRS analysis for NCR cohort | | | | | | | |
| Threshold | N | BETA | SE | OR | CI | *P-values* | *FDR q-values* |
| 5.00E-08 | 465 | 0.063 | 0.103 | 1.07 | 0.870-1.30 | 0.5411 | 0.5556 |
| 1.00E-05 | 465 | 0.061 | 0.103 | 1.06 | 0.868-1.30 | 0.5556 | 0.5556 |
| 1.00E-04 | 465 | 0.108 | 0.102 | 1.11 | 0.912-1.36 | 0.2907 | 0.3780 |
| 0.001 | 465 | 0.117 | 0.102 | 1.12 | 0.920-1.37 | 0.2534 | 0.3661 |
| 0.005 | 465 | 0.099 | 0.102 | 1.10 | 0.903-1.35 | 0.3348 | 0.3956 |
| 0.01 | 465 | 0.139 | 0.103 | 1.15 | 0.939-1.40 | 0.1774 | 0.2883 |
| 0.05 | 465 | 0.234 | 0.105 | 1.26 | 1.028-1.55 | 0.0264 | 0.0490 |
| 0.1 | 465 | 0.308 | 0.106 | 1.36 | 1.107-1.67 | 0.0035 | 0.0076 |
| 0.2 | 465 | 0.336 | 0.107 | 1.40 | 1.134-1.73 | 0.0017 | 0.0045 |
| 0.3 | 465 | 0.358 | 0.107 | 1.43 | 1.160-1.76 | 0.0008 | 0.0036 |
| 0.4 | 465 | 0.347 | 0.107 | 1.41 | 1.146-1.75 | 0.0012 | 0.0040 |
| 0.5 | 465 | 0.366 | 0.108 | 1.44 | 1.168-1.78 | 0.0007 | 0.0036 |
| 1 | 465 | 0.377 | 0.108 | 1.46 | 1.181-1.80 | 0.0005 | 0.0036 |
| PRS analysis for NHGRI cohort | | | | | | | |
| Threshold | N | BETA | SE | OR | CI | *P-values* | *FDR q-values* |
| 5.00E-08 | 1371 | 0.080 | 0.061 | 1.08 | 0.960-1.22 | 0.1928 | 0.2278 |
| 1.00E-05 | 1371 | 0.111 | 0.062 | 1.12 | 0.990-1.26 | 0.0718 | 0.1167 |
| 1.00E-04 | 1371 | 0.087 | 0.062 | 1.09 | 0.965-1.23 | 0.1642 | 0.2278 |
| 0.001 | 1371 | 0.012 | 0.061 | 1.01 | 0.898-1.14 | 0.8493 | 0.8493 |
| 0.005 | 1371 | 0.041 | 0.061 | 1.04 | 0.924-1.17 | 0.5025 | 0.5444 |
| 0.01 | 1371 | 0.082 | 0.062 | 1.09 | 0.962-1.22 | 0.1822 | 0.2278 |
| 0.05 | 1371 | 0.136 | 0.062 | 1.15 | 1.014-1.29 | 0.0293 | 0.0706 |
| 0.1 | 1371 | 0.168 | 0.062 | 1.18 | 1.047-1.34 | 0.0070 | 0.0706 |
| 0.2 | 1371 | 0.141 | 0.063 | 1.15 | 1.018-1.30 | 0.0247 | 0.0706 |
| 0.3 | 1371 | 0.132 | 0.063 | 1.14 | 1.009-1.29 | 0.0349 | 0.0706 |
| 0.4 | 1371 | 0.136 | 0.063 | 1.15 | 1.013-1.30 | 0.0303 | 0.0706 |
| 0.5 | 1371 | 0.141 | 0.063 | 1.15 | 1.017-1.30 | 0.0254 | 0.0706 |
| 1 | 1371 | 0.130 | 0.063 | 1.14 | 1.007-1.29 | 0.0380 | 0.0706 |

| **Table S6.** GO pathways enriched with genes associated to ADHD in the NHGRI Family Cohort | | | | |
| --- | --- | --- | --- | --- |
| Pathway | *P-values* | *FDR q-values* | Description | Trait |
| GO:0038187 | 2.16E-06 | 0.00061006 | pattern recognition receptor activity | ADHD |
| GO:0032451 | 0.00032581 | 0.04593976 | demethylase activity | ADHD |
